# Evaluation of tangential flow filtration coupled to long-read sequencing for ostreid herpesvirus type 1 genome assembly

**DOI:** 10.1099/mgen.0.000895

**Published:** 2022-11-10

**Authors:** Aurélie Dotto-Maurel, Camille Pelletier, Benjamin Morga, Maude Jacquot, Nicole Faury, Lionel Dégremont, Maëlis Bereszczynki, Jean Delmotte, Jean-Michel Escoubas, Germain Chevignon

**Affiliations:** ^1^​ Ifremer, ASIM, F-17390 La Tremblade, France; ^2^​ IHPE, Univ. Montpellier, CNRS, Ifremer, UPVD, F-34095 Montpellier, France

**Keywords:** *Crassostrea gigas*, Illumina, ostreid herpesvirus type 1, oxford nanopore technologies, tangential flow filtration, virus

## Abstract

Whole-genome sequencing is widely used to better understand the transmission dynamics, the evolution and the emergence of new variants of viral pathogens. This can bring crucial information to stakeholders for disease management. Unfortunately, aquatic virus genomes are usually difficult to characterize because most of these viruses cannot be easily propagated *in vitro*. Developing methodologies for routine genome sequencing of aquatic viruses is timely given the ongoing threat of disease emergence. This is particularly true for pathogenic viruses infecting species of commercial interest that are widely exchanged between production basins or countries. For example, the ostreid herpesvirus type 1 (OsHV-1) is a Herpesvirus widely associated with mass mortality events of juvenile Pacific oyster *Crassostrea gigas*. Genomes of Herpesviruses are large and complex with long direct and inverted terminal repeats. In addition, OsHV-1 is unculturable. It therefore accumulates several features that make its genome sequencing and assembly challenging. To overcome these difficulties, we developed a tangential flow filtration (TFF) method to enrich OsHV-1 infective particles from infected host tissues. This virus purification allowed us to extract high molecular weight and high-quality viral DNA that was subjected to Illumina short-read and Nanopore long-read sequencing. Dedicated bioinformatic pipelines were developed to assemble complete OsHV-1 genomes with reads from both sequencing technologies. Nanopore sequencing allowed characterization of new structural variations and major viral isomers while having 99,98 % of nucleotide identity with the Illumina assembled genome. Our study shows that TFF-based purification method, coupled with Nanopore sequencing, is a promising approach to enable in field sequencing of unculturable aquatic DNA virus.

## Data Summary

All sequence data have been deposited in NCBI under Bioproject ID PRJNA823871. Nanopore data have been deposited to SRA under accession number SAMN27363714 with base-called fastq reads under accession number SRR19025776 and raw fast5 and slow5 file under accession number SRR19039499; Illumina fastq reads have been deposited to SRA under accession number SAMN27363675. OsHV-1 assembled genome from Nanopore data have been deposited to GenBank under accession number ON411413; OsHV-1 assembled genome from Illumina data have been deposited to GenBank under accession number ON411412.

**Impact Statement**

Several aquatic viruses are pathogenic to species of commercial interest, leading to massive value losses during disease outbreaks. To better understand the origin, transmission and propagation of such viruses, more genomic data is required and needs to be analysed. However genomic characterization of viruses that are mainly unculturable is a major challenge. In the present study, we have successfully tested the combination of tangential flow filtration (TFF) and Nanopore sequencing for virus purification and genome sequencing. Our results demonstrate that it is now possible to easily sequence the complete genome of large DNA viruses directly from host infected tissues without prior *in vitro* propagation.

## Introduction

Viruses found in aquatic environments are often unculturable and their genomic characterization often relies on our ability to enrich samples for viral nucleic acid. To date, several methods have been implemented to this end and different workflows are applied depending on the type of virus studied: small or large virus, RNA or DNA virus, virus with linear or circular genome, single or double strand virus. Enrichment methods range from rapid and simple steps involving low-speed centrifugation, filtration and nuclease treatment, to more complex methods involving ultracentrifugation, tilling PCR, rolling circle amplification and hybridization-based capture. While the former shows higher efficiency at purifying viral nucleic acid, these methods habitually rely on *a priori* knowledge of the targeted virus and are limited at detecting a new variant or new pathogen [1–4]. Recently, metagenomic next-generation sequencing (mNGS) has been successfully tested for broad-spectrum pathogen identification for clinical diagnosis of infectious diseases, outbreak surveillance, and pathogen discovery [5, 6]. However, the cost, sequencing depth and background contamination are true limitations to the accuracy of mNGS-based diagnostics relative to specific targeting testing.

Given that aquaculture is continuously faced with the challenge of identifying novel pathogenic viruses infecting different fish, crustaceans, and shellfish species, there is an urgent need to develop tools enabling those viruses to be sequenced easily and with limited bias to develop timely disease control strategies.

Ostreid herpesvirus type 1 (OsHV-1) is a double strand DNA virus belonging to the Malacoherpesviridae family and is the only species of the genus Ostreavirus [7]. OsHV-1 has been associated with mass mortality events of naive Pacific oyster *Crassostrea gigas* [8] from larvae to adults [9, 10]. It has plagued the oyster production worldwide, from Europe to north and south America, Australia and New Zealand, and in a lesser extent in Asia, for more than a decade [11–17]. OsHV-1 mostly affects spat and juvenile (less than 18 months) in oyster farms, and leads to mass mortalities that can reach 100 % within days [18–20]. Research efforts have revealed a series of factors contributing to the disease, including seawater temperature, oyster age and genetics [8, 9, 16, 18, 21–24]. Recently, holistic molecular approaches uncovered the pathogens associated to Pacific oyster mortality syndrome (POMS) [25]. These studies showed that an infection by OsHV-1 is the critical step of the infectious process leading to an immune-compromised state followed by a microbiota destabilization that ‘opens the door’ to opportunistic bacterial pathogens (e.g. *Vibrio* spp.) that target haemocytes to induce their lysis [26]. The infectious process is completed with subsequent bacteraemia ultimately leading to oyster death (reviewed in [27]).

Since the characterization of the emergent genotype OsHV-1 µVar, associated with the 2008 high mortality events [18], several variants of the µVar genotype have been described (reviewed in [28]). However, most studies on OsHV-1 genetic diversity have been based on PCR molecular markers focusing on a few variable regions of the viral genome, which do not reflect the whole genomic diversity [29, 30]. Indeed, OsHV-1 µVar whole-genome sequencing [31] and its comparison with the reference genome published in 2005 [7] showed that both genomes differed at the nucleotide level (single nucleotide polymorphism, SNP) as well as at the structural level by the loss or gain of several genomic regions carrying many open reading frames (ORFs). This observation indicates that whole-genome sequencing is needed to fully reveal viral diversity and better understand the structure and evolution of the virus populations [31].

OsHV-1 particles harbour a linear genome of approximately 207 kilobases (kb) with the majority of the 128 ORFs detected (54.7 %), having no known homologies with other organisms [31]. OsHV-1 genomic organization is characteristic of other Herpesviruses consisting of two invertible unique regions (U_L_ for ‘Unique long’ 170 kbp; U_S_ for ‘Unique small’ 3.5 kbp) each flanked by tandem and inverted repeats (TR_L_/IR_L_: 7.5 kbp; TR_S_/IR_S_: 9.7 kbp) separated by a third unique region (X: 1.5 kbp) with a global genomic organization TR_L_–U_L_–IR_L_–X–IR_S_–U_S_–TR_S_. Furthermore, several isomers of the virus cohabit at equimolar levels in the same samples [7].

To date, 50 fully assembled OsHV-1 genomes are available in public databases, those genomes have been sequenced and assembled using various protocol and sequencing technologies (Table S1, available in the online version of this article). Although recent genomes have been obtained from short-read mNGS [31–34], OsHV-1 complete genome assembly remains a complex process. Indeed, a short-read approach is ideal for the identification of small-scale variation like SNPs but this technology struggles to identify variations at a larger scale like structure variations (SV) or large inverted and repeated regions. Due to its intrinsic Herpesvirus genome architecture with high G+C content regions, long repeated regions (TR_L_/IR_L_ and TR_S_/IR_S_), and the presence of four major isomers differing in the relative orientations of the two unique regions, it is extremely difficult, if not impossible, to solve the complete structure of OsHV-1 genome using short-read sequencing [35].

These limitations can now be addressed by long-read sequencing approaches of which Oxford Nanopore Technologies (ONT) is one example. Indeed, ONT has already demonstrated its ability to sequence and assemble several types of viruses from RNA [36–42] as well as DNA [43–46] matrices. Nanopore sequencing has also been successfully tested on Herpesviruses like the Human Herpes Virus Type 1 (HHV-1) [47], the Human cytomegalovirus (HCMV) [48], the Equine herpesvirus 1 (EHV-1) [49] and the koi herpesvirus (KHV) [50]. In addition, ONT already allowed researchers to sequence viruses infecting aquaculture species like the White spot syndrome virus (WSSV) infecting shrimp [51] or the Salmonid alphavirus (SAV) infecting Atlantic salmon [52]. Finally, ONT has also demonstrated efficiency to sequence viruses from sea-water samples [53–55]. However, it has never been tested for the characterization of the OsHV-1 genome.

In order to implement ONT long-read sequencing, high molecular weighted DNA is required in a large quantity. However, due to the lack of a cell line, that could be used for the propagation and isolation of viral particles, OsHV-1 must be extracted from infected animal tissues. Therefore, it is extremely challenging to obtain viral DNA in sufficient quantity to perform long-read mNGS.

To overcome these difficulties, we need a new approach for viral particle purification coupled with long-read sequencing to assemble complex OsHV-1 genomes efficiently and accurately.

One approach to purify viral particles is tangential flow filtration (TFF). This method has been widely used for purification of a large variety of virus particles, such as particles of human influenza A virus [56], insect baculovirus [57, 58], HIV-like virus [59], oncolytic measles virus [60] and of several waterborne viruses like norovirus and adenovirus [61]. While TFF has been used to reduce oyster pathogen concentration in seawater to bio-secure shellfish farms [62], it has never been used with the goal of purifying those pathogens. Recently, TFF coupled with Nanopore sequencing, has been used to concentrate a large amount of seawater for the characterization of bacteriophage population [63].

In the present study, we have developed a TFF-based method allowing purification and concentration of OsHV-1 infectious particles from infected oyster tissues and we have evaluated the relative benefits of long-read (Nanopore) and short-read (Illumina) sequencing for genome assembly. Our results show that TFF purification of OsHV-1 viral particles coupled with ONT sequencing is a promising strategy for OsHV-1 whole-genome reconstruction and to decipher genetic diversity and evolution of virus populations.

## Methods

### Oyster production

Experimental *C. gigas* spat was produced at the Ifremer hatchery in La Tremblade, (Marennes-Oléron Bay, Charente-Maritime, France). Oysters belonged to a line that had been characterized for its higher susceptibility to OsHV-1 [64]. Thirty adult oysters were conditioned in seawater at 20 °C for 2 months to favour gametogenesis. Then, ripe oysters were triggered for a mass spawning event in March 2015. Larvae and spat were cultured as described by [64, 65] and they were always maintained in our controlled facilities using filtered and UV-treated seawater to protect from pathogens naturally present in the Marennes-Oléron Bay. Prior to the experiment, spat (2 g and 4 cm) were acclimatized at 19 °C for 2 weeks in flow-through 120 l tanks using filtered and UV-treated seawater enriched in phytoplankton (*Skeletonema costatum, Isochrisis galbana* and *Tetraselmis suecica*).

### Viral inoculum preparation and OsHV-1 quantification

OsHV-1 inoculum were prepared from tissue of infected oysters following protocols of Schikorsky *et al.* [66]. Briefly, gills and mantle were dissected from moribund oysters naturally or experimentally infected with OsHV-1 in previous trials and frozen at −80 °C. Then, ten volumes of 0.2 µm filtered and artificial seawater (ASW) were added and tissues were crushed on ice using an Ultraturax mixer (3×5 s). The tissue mash was clarified by centrifugation (1000 ***g***, 5 min, 4 °C), and then filtered consecutively in sterile conditions using syringe filters at 8, 0.45 and 0.22 µm pore sizes (Millipore; Billerica, USA).

Quantification of OsHV-1 was carried out using quantitative PCR (qPCR) using primers amplifying a 197 bp fragment of the OsHV-1 DNA polymerase (DP) gene. DNA was extracted from viral inoculum using the QiaAmp DNA Mini Kit (Qiagen) according to the manufacturer’s protocol. DNA purity (1.8 ≤ A260 nm/280 nm ≤ 2.0; 2.0 ≤ A260 nm/230 nm ≤ 2.20) and concentration were checked using a Nano-Drop ND-1000 spectrometer (Thermo Scientific) and Qubit® dsDNA HS assay kits (Molecular Probes Life Technologies), respectively.

qPCRs were performed in duplicate using a Mx3005 P Thermocycler (Agilent). Amplification reactions were each performed in a total volume of 20 µl. Each reaction contained 5 µl of DNA extracted from viral inoculum or from TFF fractions (readjusted to initial inoculum volume) or 5 µl of Milli-Q water for the negative control, 10 µl of Brilliant III Ultra-Fast SYBR® Green PCR Master Mix (Agilent), 2 µl of primer OsHVDP For (forward) 5′-ATTGATGATGTGGATAATCTGTG-3′ and 2 µl of primer OsHVDP Rev (reverse) 5′-GGTAAATACCATTGGTCTTGTTCC-3′ [67] at the final concentration of 550 nM each, and 1 µl of Milli-Q water. qPCR cycling conditions were as follows: 3 min at 95 °C followed by 40 cycles of amplification at 95 °C for 5 s and 60 °C for 20 s. For absolute quantification, the amplification product of the DP gene was cloned into the pCR4-Topo vector and replicated in *Escherichia coli* DH5α (Invitrogen). Plasmid was extracted using the Wizard Plus SV miniprep DNA purification system (Promega) and standard curves of known concentration of plasmid were generated according to the Applied Biosystems manual for absolute real-time PCR quantification. Absolute quantification of OsHV-1 genome copies in oyster samples was then estimated by comparing the observed crossing point (Cp) values to known plasmid standards from 10^3^ to 10^9^ copies of the DP product. The results were expressed as the log-transformed copy number of viral DNA per micro litter of DNA extracts (cp μl^−1^).

### Virus-like particle quantification by epifluorescence microscopy

Virus-like particles (VLP) were quantified using epifluorescence microscopy after staining with nucleic acid dye [68, 69]. Briefly, viral inoculum were first fixed with glutaraldehyde (Sigma-Aldrich G6257, final concentration 0.5 %) and diluted in 1 % potassium citrate solution (pH 7) to prevent aggregation of viral particles. Inoculum were vacuum filtrated to capture viruses on a 0.02 µm aluminium oxide filter (Whatman 6809–6002 Anodisc 25 inorganic membrane filter), before subsequent staining (SYBR Gold nucleic acid gel stain Invitrogen S11494, diluted at 0.2 % in Tris-EDTA) and washing by 4 ml of 1 % potassium citrate solution. Filters were then mounted on glass slides and VLP were observed and enumerated under epifluorescence microscopy (Olympus BH2-RFCA).

### Experimental infections

For experimental infections, oysters were previously myorelaxed in hexahydrate MgCl2 (ACROS, 50 g l^−1^) [70]. Oysters were injected using 26-gauge needle attached to a multi-dispensing hand pipette, with 100 µl of inoculum (diluted in ASW to reach the desired viral concentration) in the abductor muscle. For each experiment, a group of oysters was myorelaxed and injected with ASW as the control. Oysters were replaced in seawater in plastic tanks at 20 °C and mortality was monitored daily.

### Virus purification and concentration by tangential flow filtration (TFF)

To develop a TFF-based process for the concentration of OsHV-1 particles, we used MicroKros® hollow fibre filter modules (Spectrum®) with three different pore sizes: 750 kDa (CO2-E7500-05-N), 500 kDa (CO2-E500-05-N) and 300 kDa (CO2-E300-05-N).

**Fig. 1.**
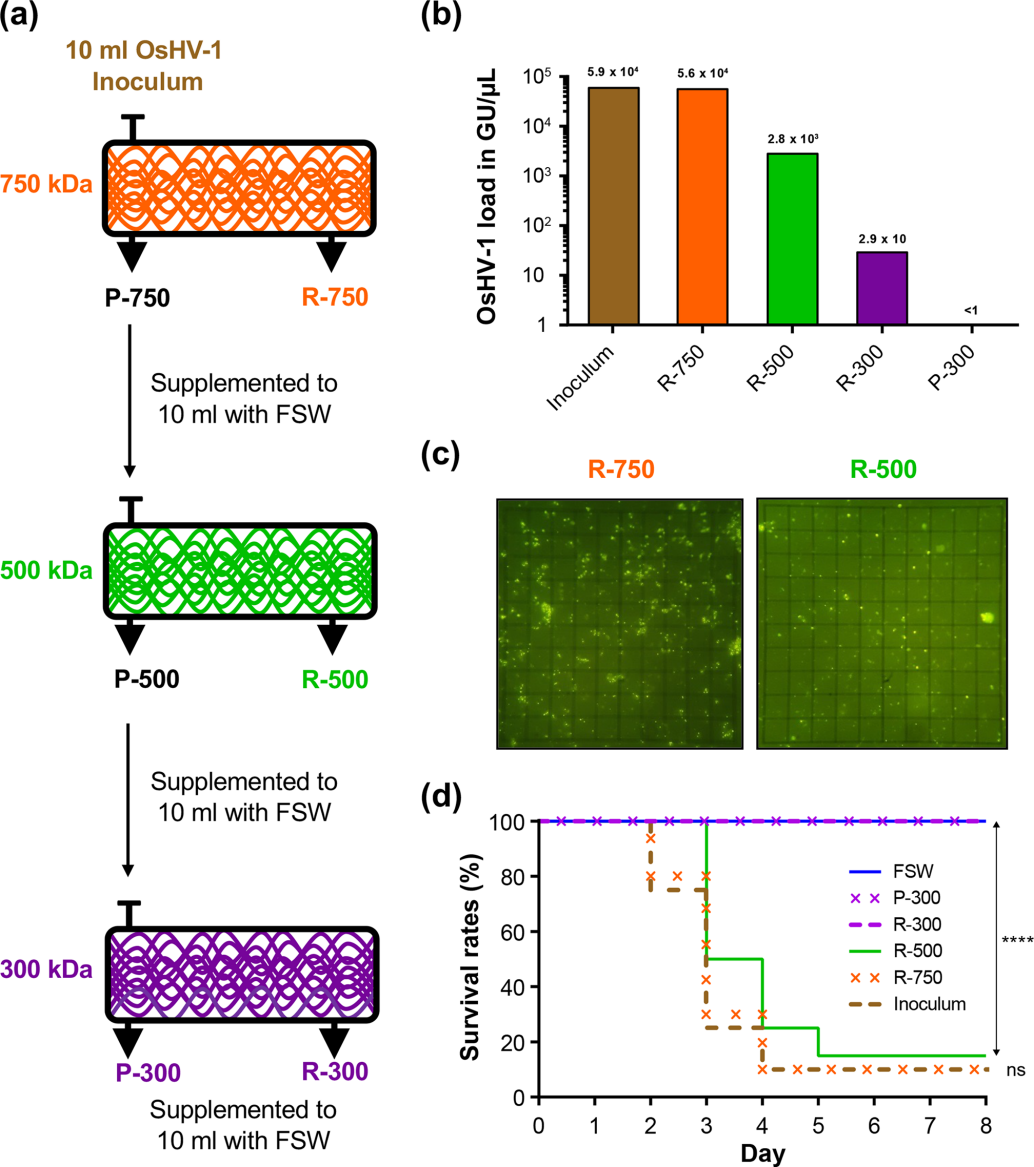
(a) Workflow for the processing of OsHV-1 inoculum on tangential flow filtration (TFF) devices with three membrane pore sizes (750, 500 and 300 kDa). After filtration on each TFF-device, the volumes of retentates (R) and permeates (P) were supplemented to 10 ml with filtered seawater (FSW, 0,02 µm). (b) qPCR monitoring of OsHV-1 loads (in genomic units per µl) at the different steps of TFF. (c) Visualization and quantification of virus-like particles (VLP) by epifluorescence microscopy in retentates of TFF devices of 750 kDa (R-750) and 500 kDa (R-500). An eyepiece with a 10×10 grid reticle was used for counting VLP (note in our setup the grid reticle is 100×100 µm). (d) Kaplan-Meier survival curves of oysters injected with 100 µl of OsHV-1 inoculum, or retentates of TFF-devices of 750, 500, 300 kDa (R-750, R-500 and R-300, respectively), or permeate of TFF device 300 kDa (P-300) or FSW as negative control. ****P <0.0001; ns not significant (Mantel–Cox Log-rank test).

The first step of the process was to determine the pore size of the appropriate membrane to retain the OsHV-1 particles. To this end, we have filtered 10 ml of OsHV-1 inoculum containing 5.9×10^4^ genomic units (GU)/µl with the 750 kDa TFF-device until the volume reached 1 ml. This 1 ml retentate (R), consisting of particles larger than the pore size of the hollow fibre, was diluted to the initial volume (10 ml) with filtered (0.02 µm) seawater and stored at 4 °C. The permeate (P), composed of particles smaller than the pore size of the hollow fibre, was collected, the volume adjusted to 10 ml with filtered (0.02 µm) seawater and was then filtered on the 500 kDa TFF device. The 500 kDa retentate was then processed similarly to the 750 kDa retentate and the 500 kDa permeate was then filtered on the 300 kDa TFF-device. At the end of this filtering process, we obtained three retentates (R-750; R-500 and R-300) and one permeate (P-300) (Fig. 1a).

The second step consisted of purifying and concentrating OsHV-1 particles to extract enough DNA for both short- and long-read sequencing. For this purpose, we prepared a viral inoculum (see Viral inoculum preparation and OsHV-1 quantification for details) from 31.12 g of tissue obtained from 28 susceptible oysters (family F15) naturally infected by OsHV-1 in the Thau Lagoon in 2016 and stored at −80 °C (for more details on oyster origin and infection process see [71]). We obtained 462 ml of 0.22 µm filtrated inoculum, which were processed on a 500 kDa hollow fibre filter. Concentrated viral particles contained in 82 ml of the 500 kDa retentate were recovered by centrifugation (2 h at 18 000 r.p.m. at 4 °C). Pellets were then re-suspended in 1.2 ml of PBS and DNA was extracted and quantified as described above. We finally obtained 17.9 µg of viral DNA.

### Illumina short-read sequencing

Illumina DNA-Seq library preparation and sequencing were performed by the Genome Quebec Company (Genome Quebec Innovation Center, McGill University, Montreal, Canada) using 120 ng of DNA extracted from the OsHV-1 inoculum concentrated on 500 kDa TFF-device [as described at the end of Virus purification and concentration by tangential flow filtration (TFF)], the NxSeq AmpFREE Low DNA Library Kit (Lucigen) and the NovaSeq 6000 Sequencing system (Illumina®) (paired ends, 150 bp).

### Nanopore long-read sequencing

For long-read sequencing, we used the Oxford Nanopore Technologies (ONT) MinION platform (Mk1b). Briefly, 10 µg of DNA extracted from the OsHV-1 inoculum concentrated on 500 kDa TFF-device [as described at the end of Virus purification and concentration by tangential flow filtration (TFF)] were fractionated on Agencourt AMPure XP beads (Beckmann Coulter, 0.5 × final concentration) to eliminate small DNA molecules (< 300 bp). DNA size range analysis on Agilent Fragment Analyzer (Genomic DNA analysis kit DNF-467–33) revealed that the average size was over 60 kb with a genomic quality number (GQN) of 8.9 (threshold 10 kb). Then, 400 ng of AMPure purified DNA were used to construct the sequencing library using SQK-RAD0004 tagmentation kit and sequencing was performed as per manufacturer’s guidelines using R9.4 flowcells (FLO-MIN106) and run for 20 h.

### Illumina genome assembly

**Fig. 2.**
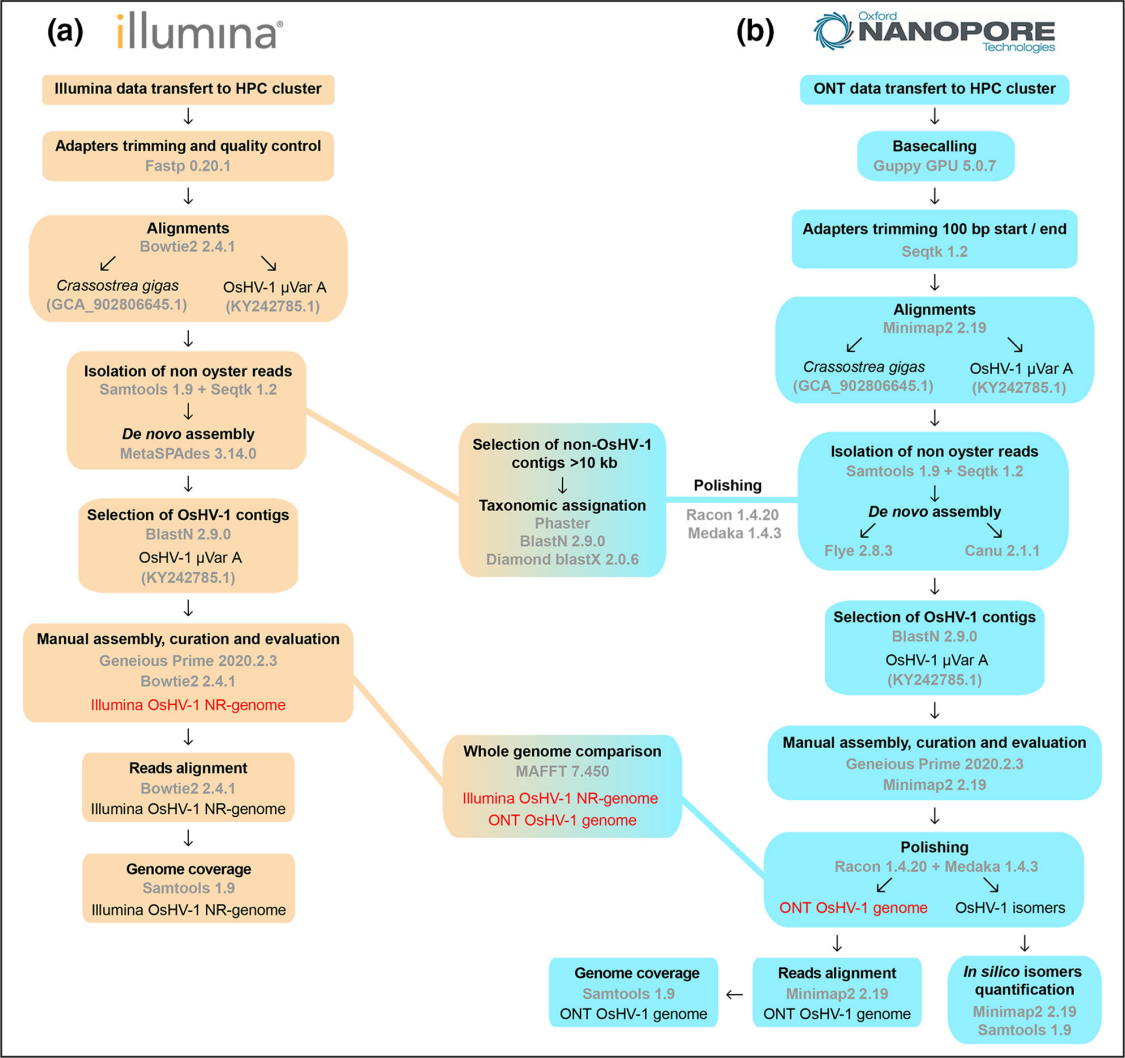
Flow diagram to illustrate the bioinformatic pipelines used to assemble OsHV-1 genome from (a) Illumina short-read or (b) Nanopore long-read.

Illumina raw reads files were quality filtered (PHRED score >31) and adapters trimmed with Fastp version 0.20.1 [72]. To evaluate the origin of reads, fastq reads were aligned to the oyster *C. gigas* genome (accession: GCA_902806645.1) and to OsHV-1 µVar A genome (accession: KY242785.1) with Bowtie2 version 2.4.1 [73]. All the non-oyster reads were then selected with Samtools version 1.9 [74] and Seqtk version 1.2 (https://github.com/lh3/seqtk). Those reads were then used for *de novo* assembly with SPAdes version 3.14.0 with parameter –meta [75]. The *de novo* OsHV-1 contigs generated by SPAdes were then identified with blastn version 2.6.0 [76] using OsHV-1 µVar A sequence as a query (accession number: KY242785.1). The identified OsHV-1 contigs were then manually scaffolded in Geneious Prime (version 2020.2.3) to reconstruct the common virus architecture (U_L_-IR_L_-X-IR_S_-U_S_) [7]. To overcome the problem of inverted repeated regions that cannot be confidently positioned with short-read data, we constructed non-redundant genomes (NR genomes) that contain only one copy of each repeated region (U_L_-IR_L_-X-IR_S_-U_S_) an approach commonly used for Herpesvirus genome analyses [34, 77, 78]. Quality filtered reads were then aligned to the new constructed OsHV-1 NR genome to evaluate coverage uniformity. This pipeline is summarized in Fig. 2a.

### Nanopore genome assembly

Nanopore fast5 file were GPU base called with Guppy version 5.0.7 with standard parameters except -config dna_r9.4.1_450bps_hac.cfg (https://community.nanoporetech.com/downloads). Fastq reads were then 100 bp trimmed at their beginning and at their end to ensure proper adapter removal with SeqTk version 1.3 with parameters -b 100 and -e 100 (https://github.com/lh3/seqtk). To assign reads to either host or viral origin, fastq reads were aligned to the oyster *C. gigas* genome (accession: GCA_902806645.1) and to OsHV-1 µVar A genome (accession: KY242785.1) with Minimap2 version 2.22 with standard parameters [79]. Then two types of *de novo* assemblies were performed with non-oyster reads with two different *de novo* assemblers. All fastq reads or only the top 10 % of the longest reads were used as input for *de novo* assembly with Flye version 2.8.3 and parameters -meta [80] or with Canu version 2.1.1 and parameters -nanopore [81]. OsHV-1 *de novo* assembled contigs were then identified with blastn [76] against the OsHV-1 µVar A genome and were manually trimmed in Geneious version 2020.2.4 (https://www.geneious.com). All *de novo* contigs plus the new assembled OsHV-1 genome were then polished using Nanopore reads with three run of Racon version 1.4.20 with parameters --no-trimming -m 8x−6 g −8 w 500 [82] and one run of Medaka version 1.4.3 with parameters medaka_consensus (https://github.com/nanoporetech/medaka).

For OsHV-1 isoform coverage evaluation, the four contigs produced by Canu with the top 10 % of the longest reads were trimmed on the U_L_ side to keep a 10 kb U_L_ fragment with Geneious version 2020.2.4 (https://www.geneious.com). The four major isomers were then used as a reference for reads mapping with Minimap2 version 2.22 with standard parameters -axe map-ont (https://github.com/lh3/minimap2). Samtools version 1.10 [83] were then used to filter out all the shortest aligned read and all the alignment with MapQ <40, keeping only the reads spanning at least a fragment of the U_L_ and a fragment of the U_S_ (i.e. 28 kb long). A pipeline summary can be found in Fig. 2b.

### Non-OsHV-1 contigs' classification

Polished non-OsHV-1 contigs with a size larger than 10 kb were then subjected to taxonomic identification and classification with phaster [84] accessed in September 2021, with blastn version 2.0.9 with parameters -max_hsps 1 -max_target_seqs 100 -evalue 0.0001 [76] or with diamond blastx [85] version 2.0.6 with parameters --sensitive --max-hsps 1 --max-target-seqs 0 --range-culling -F 15 --evalue 0.0001 against the refseq database accessed in September 2021.

### Comparison of Illumina and Nanopore OsHV-1 genomes

Before whole-genome alignment evaluation, both genomes were modified to fit the same isomers' version of the genome (i.e. TR_L_/U_L_->/IR_L_/X/IR_S_/U_S_->/TR_S_). To evaluate the capacity of Nanopore sequencing to produce an accurate OsHV-1 genome, the manually constructed Illumina OsHV-1 non-redundant (NR) genome was aligned with the manually curated Nanopore OsHV-1 complete genome with maftt version 1.4.0 using default parameters [86] implemented in Geneious version 2020.2.4 (https://www.geneious.com).

### OsHV-1 phylogeny

In order to illustrate the global phylogenetic relationships among our and other available genomes, 13 OsHV-1 NR-genomes (11 available in GenBank: AY509253, GQ153938, KP412538, KY242785, KY271630, MG56175, MF509813, OM811579, OM811584, OM811586, OM811594 and the two from this study) were aligned with progressiveMauve algorithm [87] implemented in Geneious version 2020.2.4 to local collinear blocks. Reordered genomes block were then aligned with MAFTT version 7.4 using default parameters [86] implemented in NGPhylogeny.fr [88]. Alignment was cleaned with BMGE with default parameters [89] implemented in NGPhylogeny.fr. The best fitting model of nucleotide substitution was chosen according to the Akaike Information Criterion with jModelTest version 2.1.10 with default parameter [90]. Tree was constructed with phyML version 3.3 [91] with default parameters except ‘-b 100 m HKY85 -f m -c 8 s BEST -a e -v e -o tlr’ implemented in NGPhylogeny.fr. Phylogenetic tree was midpoint rooted and arranged in FigTree version 1.4.4 (https://github.com/rambaut/figtree).

## Results

### Calibration of tangential flow filtration (TFF) device to purify and concentrate OsHV-1 particles

To develop a tangential flow filtration-based process for the purification and concentration of OsHV-1, we tested TFF-devices with three membrane pore sizes [molecular weight cut-off (MWCO) of 750, 500 and 300 kDa]. Then, we used an OsHV-1 inoculum as starting material and successively filtered it through the three TFF-devices (Fig. 1a; see Methods for further detail). Finally, we have obtained three retentates (R-750, R-500 and R-300) and one permeate (P-300), which were then used to analyse the OsHV-1 load and infectivity.

To evaluate the ability of the three TFF-devices to retain OsHV-1, we have quantified OsHV-1 load by qPCR in the initial inoculum, in the three retentates and in the permeate (Fig. 1b). The number of OsHV-1 genomic units (GU) in the R-750 fraction (5.6×10^4^ GU) was of the same order of magnitude as it was in the initial inoculum (5.9×10^4^ GU), which suggests that most of the viruses (95 %), were retained by 750 kDa MWCO. However, a small fraction (4.8 %) of GUs present in the initial inoculum were also found in the R-500 retentate (2.8×10^3^ GU) and traces of amplification (below standard curve) were also observed in the R-300 (2.9×10 GU). Epifluorescence microscopy did not reveal virus-like particles (VLP) in the R-300 and P-300 fractions (data not shown) while in the R-750 and R-500 fractions we were able to enumerate 2.71×10^4^ (7.05 x 10^3^±sd) and 4.88×10^3^ (2.79×10^3^±sd) VLP µl^−1^, respectively (Fig. 1c). Epifluorescence microscopy gave similar results to qPCR and confirms that most of the viral particles present in the starting inoculum were preserved in the R-750 fraction.

Finally, we compared the infectious capacity of the different fractions with the initial inoculum by injecting them into oysters. No mortality was observed for oysters injected with fractions R-300 and P-300 as well as for oysters injected with filtered sea water (Fig. 1d). However, oysters that had been injected with R-750 and R-500 fractions exhibit survival curves, which are not significantly different from those of oysters injected with the starting inoculum. Interestingly, survival curves of R-750 and R-500 fractions are not significantly different although R-500 fraction contained around 20 times less OsHV-1 GU than the R-750 fraction. These results showed that filtration on 500 kDa TFF-device allowed the purification and concentration of fully infective OsHV-1 viral particles. Consequently, we used the 500 kDa TFF-device to purify and concentrate viral particles from an inoculum prepared from 28 oysters associated from mortality event, which have occurred in 2016. We have been able to extract ~18 µg of viral DNA, an amount sufficient to perform both short- and long-read sequencing (see Methods for further detail).

### General statistics of the two sequencing technologies

Viral DNA extracted from TFF purified viral particles was sequenced using two different sequencing technologies: (i) a short-read approach based on the Illumina platform and (ii) a long-read approach based on the Nanopore platform.

The emergence of mNGS, has allowed researchers to put aside complex techniques such as ultracentrifugation coupled to sucrose gradient for OsHV-1 genome sequencing. The direct consequence of the lack of viral purification is the production of a large amount of non-viral data, resulting in the waste of a significant portion of the sequencing run. In recent studies, sequencing of the OsHV-1 genome from DNA extracted directly from infected tissues has led to the acquisition of only 0.01–13.21% OsHV-1 reads (Table S1).

**Fig. 3.**
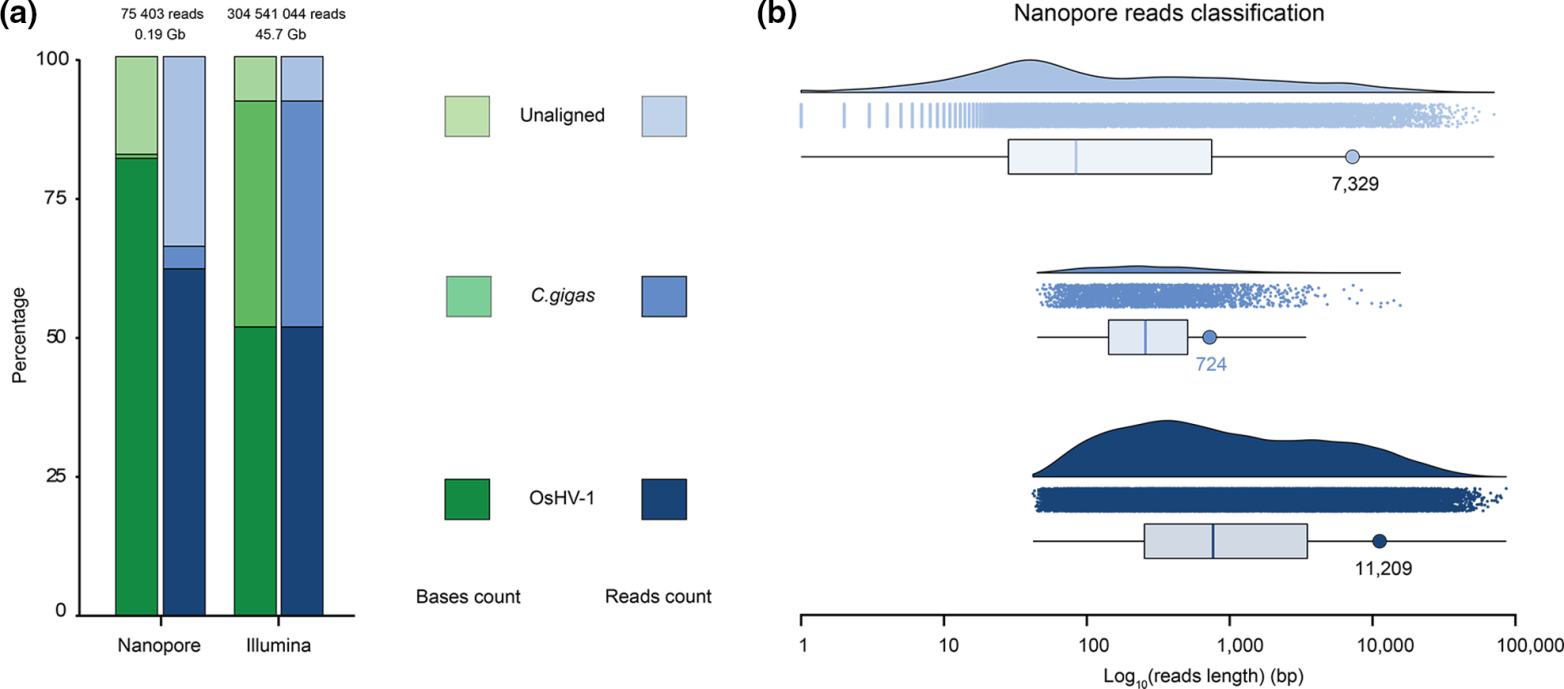
Global sequencing statistics. (a) Histogram representing percentage of sequenced bases (green) and reads (blue) that aligned to OsHV-1 (dark) or to *C. gigas* (regular) genomes or that did not align to these genomes (light). (b) Raincloud plot representing the size distribution of the Nanopore reads that aligned to OsHV-1 (dark blue) or to *C. gigas* (blue) genomes or that did not align to these genomes (light blue). Each panel is made of a density plot, a dot plot where each dot corresponds to one Nanopore read and a box plot showing average read size (vertical coloured bar) and N50 (coloured circle).

As expected TFF purification of OsHV-1 particles allowed both sequencing technologies to obtain a large enrichment in OsHV-1 reads compared to host reads, leading to a mean coverage of 740 reads/bp and 213 790 reads/bp for Nanopore and Illumina sequencing, respectively. In more details, the Illumina sequencing run yielded more than 304 million paired-end reads of 150 base pairs (bp) corresponding to 45.68 Gb and distributed as follows: 52 % of OsHV-1 reads, 40 % of *C. gigas* reads and 8 % of unaligned reads with a similar proportion of sequenced base number (Fig. 3a). The Nanopore sequencing run yielded 75 403 reads corresponding to 0,19 Gb and distributed as follows: 62 and 82 % of OsHV-1 reads and bases, respectively, 4 and 0.7 % of *C. gigas* reads and bases, respectively, and 33 and 17 % of unaligned reads and bases, respectively (Fig. 3a). Since the Nanopore technology sequence native DNA molecules present in the DNA library, a large range of reads' size has been sequenced. In detail, OsHV-1 reads ranged from 42 to 86 234 bp with a N50 of 11 209 bp, *C. gigas* reads ranged from 45 to 15 635 bp with a N50 of 724 bp and unaligned reads ranged from 1 to 70 966 bp with a N50 7 329 bp (Fig. 3b). The difference in N50 between host and viral DNA (11 209 vs 724 bp) was probably due to oyster DNA fragmentation during purification steps while viral DNA was protected by the viral envelope and capsid. This observation has probably led to the observed difference in host reads' percentage between both sequencing approaches. Indeed, before and during the Nanopore libraries' construction several Ampure XP bead-based DNA purification were performed, and this type of DNA purification is known to induce a loss of small size DNA molecules.

### OsHV-1 genome assembly using Illumina reads

**Fig. 4.**
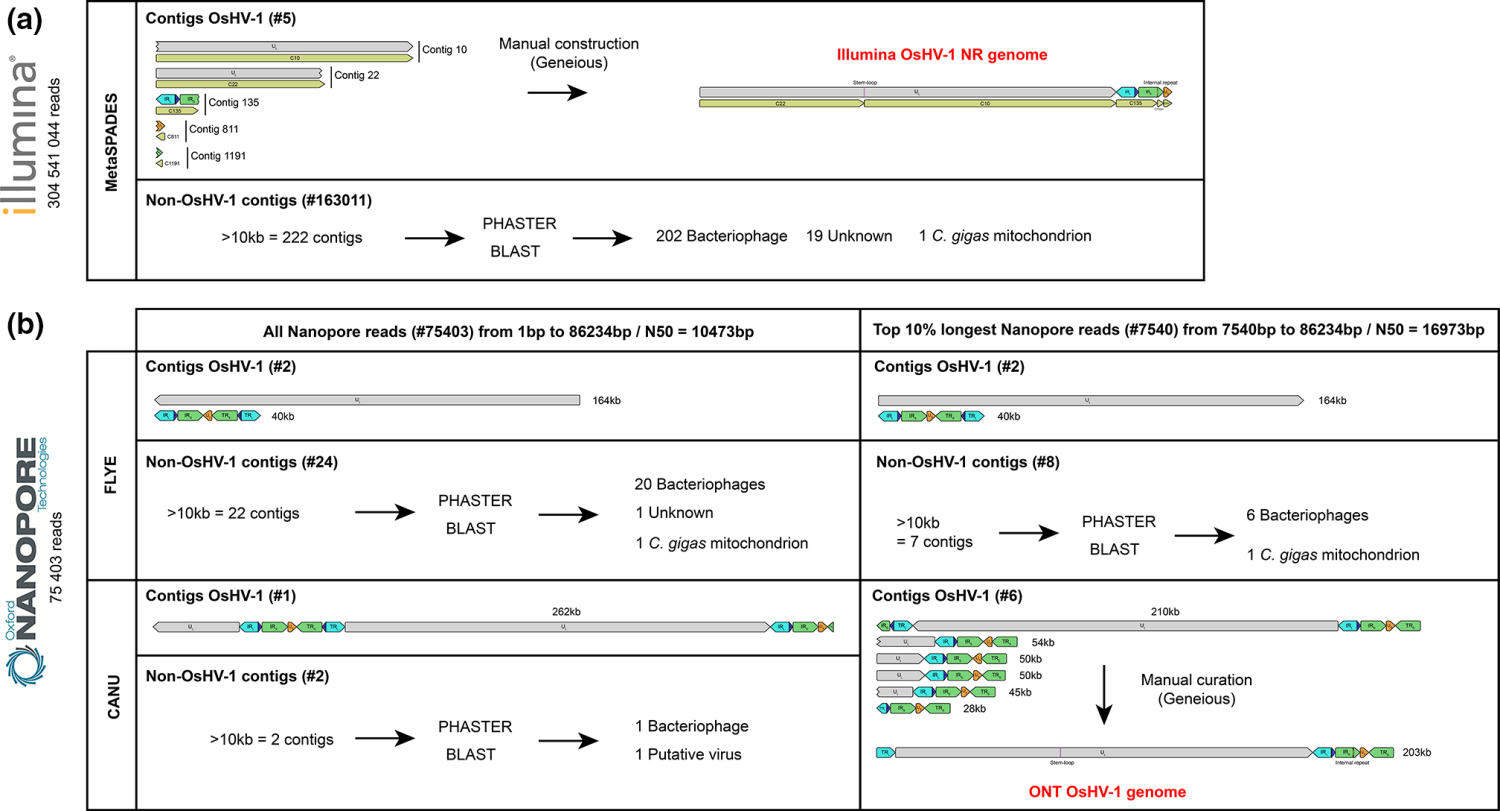
Schematic representation of OsHV-1 whole-genome assembly outputs for (a) Illumina short-read sequencing and (b) Nanopore long-read sequencing.

Our short-read pipeline based on the *de novo* assembler metaSPAdes produced five OsHV-1 contigs ranging from 2 429 to 99 197 bp (Fig. 4a). Those five contigs were then manually assembled to construct an OsHV-1 NR genome in which only one copy of each repeat was conserved (U_L_-IR_L_-X-IR_S_-U_S_, see Methods for further details). The re-alignment of the Illumina reads on the new OsHV-1 NR genome allowed us to correct the junction between contigs C1191 and C811 (addition of one A/T bp) and to confirm that the other metaSPAdes contigs were correctly assembled.

**Fig. 5.**
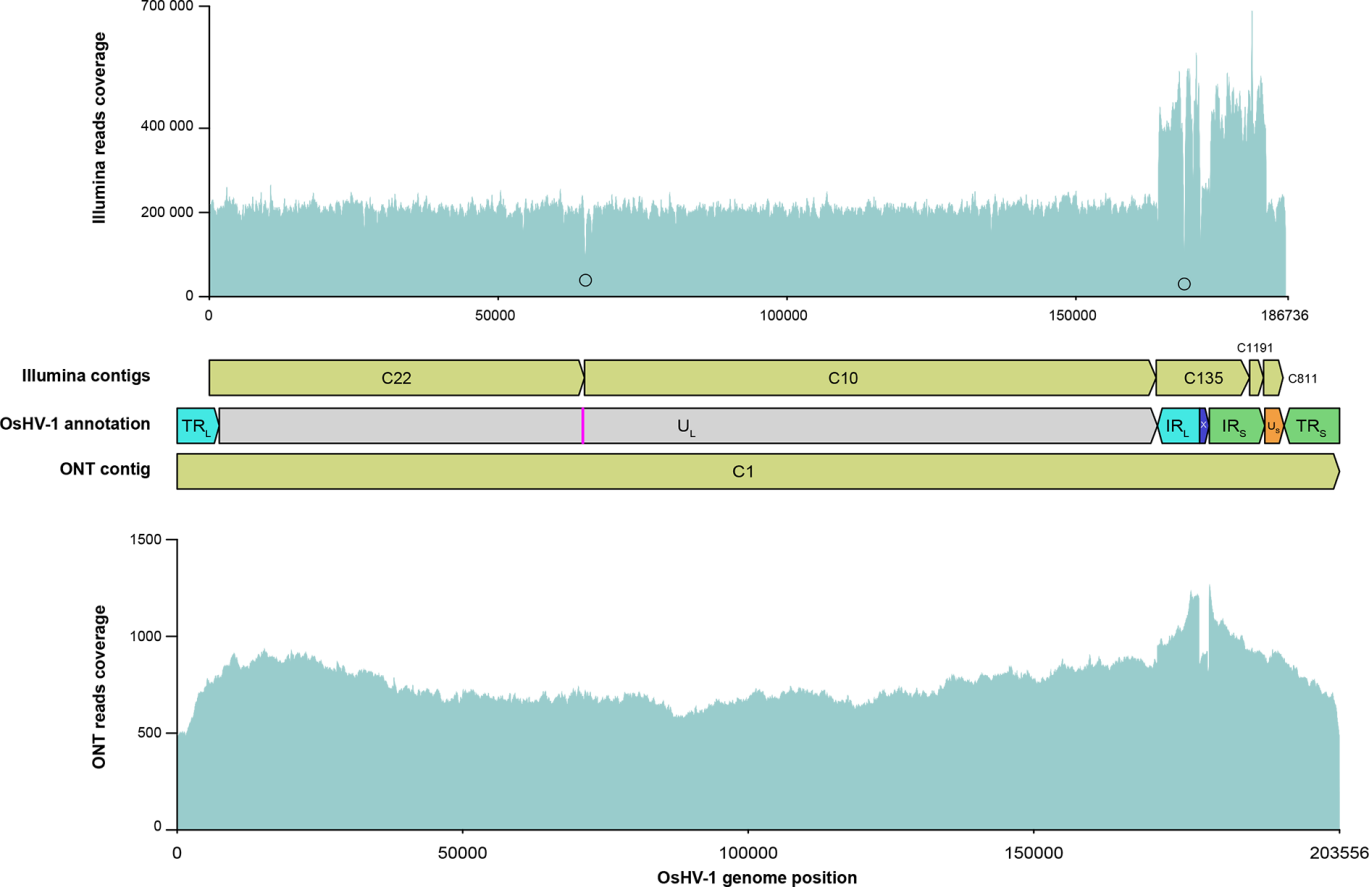
OsHV-1 whole-genome assemblies' evaluation by read mapping. Upper panel represent Illumina short-read coverage on the Illumina OsHV-1 NR genome. Black circles indicate bottom line of the two losses of coverage identified in the Illumina OsHV-1 NR genome. Middle panel represent Illumina *de novo* assembled contigs (green), OsHV-1 genomic region annotation with the pink bar representing the stem-loop and the ONT *de novo* assembled contig (green). Bottom panel represent ONT long-read coverage on the ONT OsHV-1 genome. C: Contig.

As expected, we observed that the reads' coverage within IR_L_ and the IR_S_ is twice the reads' coverage for unique regions U_L_, U_S_ and X given that the NR genome contains only one copy of theses duplicated regions (Fig. 5 upper panel). Interestingly, two decreases of reads' coverage were observed (Fig. 5 upper panel). The first decrease of coverage was at the junction of the C22 and C10 contigs and takes place in a stem-loop suspected to be the viral origin of replication [7]. The presence of this stem-loop could have induced a difficulty to access the DNA in this region by the sequencing polymerase leading to a decrease of sequencing yield around this palindromic region and the production of two distinct contigs by the *de novo* assembler (Fig. S1). The second decrease of reads' coverage takes place within the C135 contig, in a long C22 homopolymer of the IR_L_ region. This type of bias in read coverage has already been observed for long homopolymer sequenced by Illumina [92] (Fig. 5 upper panel and Fig. S2).

### OsHV-1 genome assembly using Nanopore reads

Since this is the first time that an OsHV-1 genome has been assembled from ONT long-read, we decided to evaluate the two most used long-read *de novo* assembler Flye and Canu with two datasets: (i) all the Nanopore reads and (ii) only the top 10 % longest Nanopore reads (see Methods for further detail) (Fig. 4b).

Whatever the dataset used, Flye yielded the same two contigs; a long one (164 kb) corresponding to the complete U_L_ and a short one (40 kb) made of the U_S_ surrounded by one copy of IR_L_-X-IR_L_ on its left side and one copy of TR_S_-X-TR_L_ on its right side (Fig. 4b). With the dataset including all Nanopore reads, Canu yielded one large OsHV-1 contig (262 kb). This contig is made of one fragment of the U_L_ (32 kb) followed by a fragment made of the U_S_ surrounded by one copy of IR_L_-X-IR_S_ on its left side and one copy of TR_S_-X-TR_L_ on its right side (40 kb), followed by the complete U_L_ (164 kb) and followed by another fragment made of U_S_ surrounded by one copy of IR_L_-X-IR_S_ on the left side and one copy of the TR_L_ (24 kb) on its right side (Fig. 4b).

**Fig. 6.**
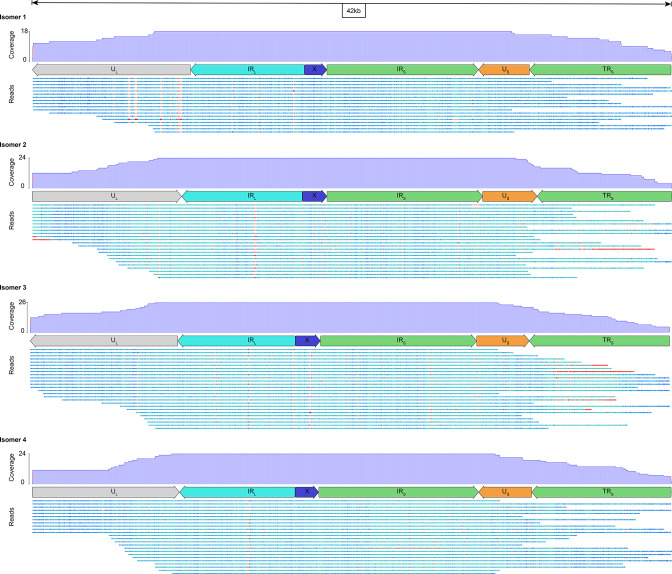
OsHV-1 major isomer validation. Each panel corresponds to a 42 kb long OsHV-1 genomic region made of U_L_ fragment plus IR_L_, X, IR_S_, U_S_ and TR_S_. The different panels correspond to the different OsHV-1 isomers made by permutation of U_L_ and U_S_ regions. Each panel is made of a diagram of reads coverage, a diagram of OsHV-1 region annotation and a diagram representing long-read alignment that span at least one fragment of the U_L_ and one fragment of the U_S_. Read bases are color-coded from conserved (dark blue), to less conserved (light blue) with gaps in sequence colored in red.

With the dataset composed of only the top 10 % longest Nanopore reads, Canu yielded six OsHV-1 contigs ranging from 28 to 210 kb (Fig. 4b). The 28 kb contig consisted of the U_S_ surrounded on its left side by one copy of TR_L_-X-IR_S_ and on its right side by one copy of TR_S_. The four contigs ranging from 45 to 54 kb share the same general structure; that is to say, a fragment of the U_L_ region followed by a copy of IR_L_-X-IR_S_-U_S_-TR_S_. These four contigs are differentiated by the relative orientation of the U_L_ and U_S_ fragments (same or inverted orientations) and their orientation with respect to the IR_L_-X-IR_S_ fragment (Fig. 4b). It was likely that these four contigs correspond to the four major OsHV-1 isomers described by Davison *et al.* [7]. In order to verify this observation and to quantify the relative abundance of the four isomers, we have screened all the reads long enough to cover at least a part of both U_L_ and U_S_ regions. We identified 92 long-read divided into 18 <-U_L_/<-U_S_, 24 U_L_->/U_S_->, 26 <-U_L_/U_S_-> and 24 U_L_->/<-U_S_ (Fig. 6). This result confirms the OsHV-1 genome isomerization and shows that the viral population is composed of approximately equimolar amounts of the four isomers. Finally, Canu assembled one large contig (210 kb) made of the complete U_L_ surrounded by one copy of IR_S_-X-TR_L_ on its left side and one copy of IR_L_-X-IR_S_-U_S_-TR_S_ on its right side (Fig. 4b). This former contig have been manually curated to create the complete ONT OsHV-1 genome by removing the IR_S_-X fragment on the left side of the U_L_ and by reverse complement the U_L_ to fit with the most used representation of OsHV-1 genomes isomer [7].

To assess the robustness of the manual assembly, we aligned all ONT reads back to the ONT OsHV-1 genome and observed a continuity of coverage throughout the genome, which validates the assembly of the ONT OsHV-1 genome with our pipeline (Fig. 5 lower panel). Interestingly, we can see a small decrease of coverage at both extremities of the genome and an increase of coverage on IR_L_ and IR_S_ at the junction surrounding the X region (Fig. 5 lower panel). This observation reflects the use of one of the 16 minor isomers described by Davison *et al.* to represent our ONT OsHV-1 genome [7]. Indeed, in addition to the four isomers described previously, Davison *et al.* showed that some of these isomers contained an additional copy of the X region at the left or right terminus whereas some might have lacked the X region internally. Since only one copy of the X region is presented in the ONT OsHV-1 genome, the reads corresponding to the minor variant of the X region might tend to align around the X region between IR_L_ and IR_S_ instead of aligning at the extremities of the genome (TR_L_ and TR_S_) leading to a higher coverage of the region surrounding X compared to the rest of the genome (Fig. 5 lower panel).

Taken together these results validates the assembly of the ONT OsHV-1 genome, confirms for the first time the OsHV-1 genome isomerization and shows that the viral population is composed of approximately equimolar amounts of the four major isomers.

### Comparison of Illumina OsHV-1 NR genome and ONT OsHV-1 genome

**Fig. 7.**
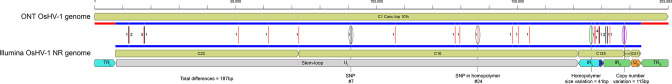
ONT and Illumina OsHV-1 whole-genome comparison. A total of 187 bp differences are identified between both genomes. Overall, 7 bp correspond to SNPs (black bar), 24 bp correspond to SNPs in small homopolymer (red bar), 41 bp correspond to a homopolymer size variation (green bar), and 115 bp correspond to a copy number variation (purple bar). Numbers next to the bars correspond to the number of variable bases at that position.

Since it is almost impossible to assemble a complete OsHV-1 genome from short-read technology due to the presence of large inverted repeats, and because long-read technology tends to produce longer OsHV-1 genome than expected, both technologies still requires manual editing to join the different fragments or to remove extra repeats to obtain a genome structure as accurate as possible. Furthermore, ONT is known to produce a high error rate on raw sequences compared to the Illumina technology. Here, rather than looking at the raw sequence error rate, we attempted to quantify the residual error by comparing to each other, the polished ONT OsHV-1 genome, and the Illumina OsHV-1 NR genome. To do so, we used a pairwise alignment and were able to identify a total of 187 bp difference between the two genomes (Fig. 7).

Those differences could be classified in three categories: (i) SNPs, (ii) copy number variation of a 115 bp repeat and (iii) large homopolymer size variation. We observed a total of 31 SNPs between the two genomes, 24 SNPs correspond to InDel, which take place at the extremity of small homopolymers (from 5 to 11 bp), and 7 SNPs were distributed elsewhere in the genome. The 115 bp copy number variation takes place into the IR_S_ region. While the Illumina OsHV-1 genome contains two copies of the 115 bp sequence, the ONT OsHV-1 genome harbour three copies of this repetition (Fig. S3). Finally, we observed an homopolymer size variation in the IR_L_ region corresponding to 41 bp insertion in the ONT OsHV-1 genome compared to Illumina OsHV-1 genome. Since sequencing error in homopolymer is a well-known caveat of Nanopore sequencing [93], and because long homopolymer are known to be error prone also in Illumina sequencing [92, 94], we have performed a careful analysis of the read alignment for both technologies in this specific region. Interestingly, alignment observation in this large homopolymer identify heterogeneity in homopolymer size at the level of individual reads for both sequencing technologies. While size variation for Illumina reads ranged from 7 to 22 bp, size variation for Nanopore reads ranged from 5 to 98 bp (Fig. S2A and B respectively).

Because of the impossibility to accurately distinguish error in this large homopolymer for both Illumina and Nanopore sequencing, and because we cannot accurately assign the number of Cs inside this large homopolymer, we have decided to keep the most represented number of Cs for each of the sequencing techniques. We therefore did not consider this large homopolymer in our final pairwise homology comparison of both sequencing technologies.

Finally, if we disregard the additional 115 bp repeat highlighted by ONT sequencing, the two *de novo* assembled genomes only differ by a total of 31 bp and show 99.98 % of nucleotide identity.

### OsHV-1 phylogenetic analysis

**Fig. 8.**
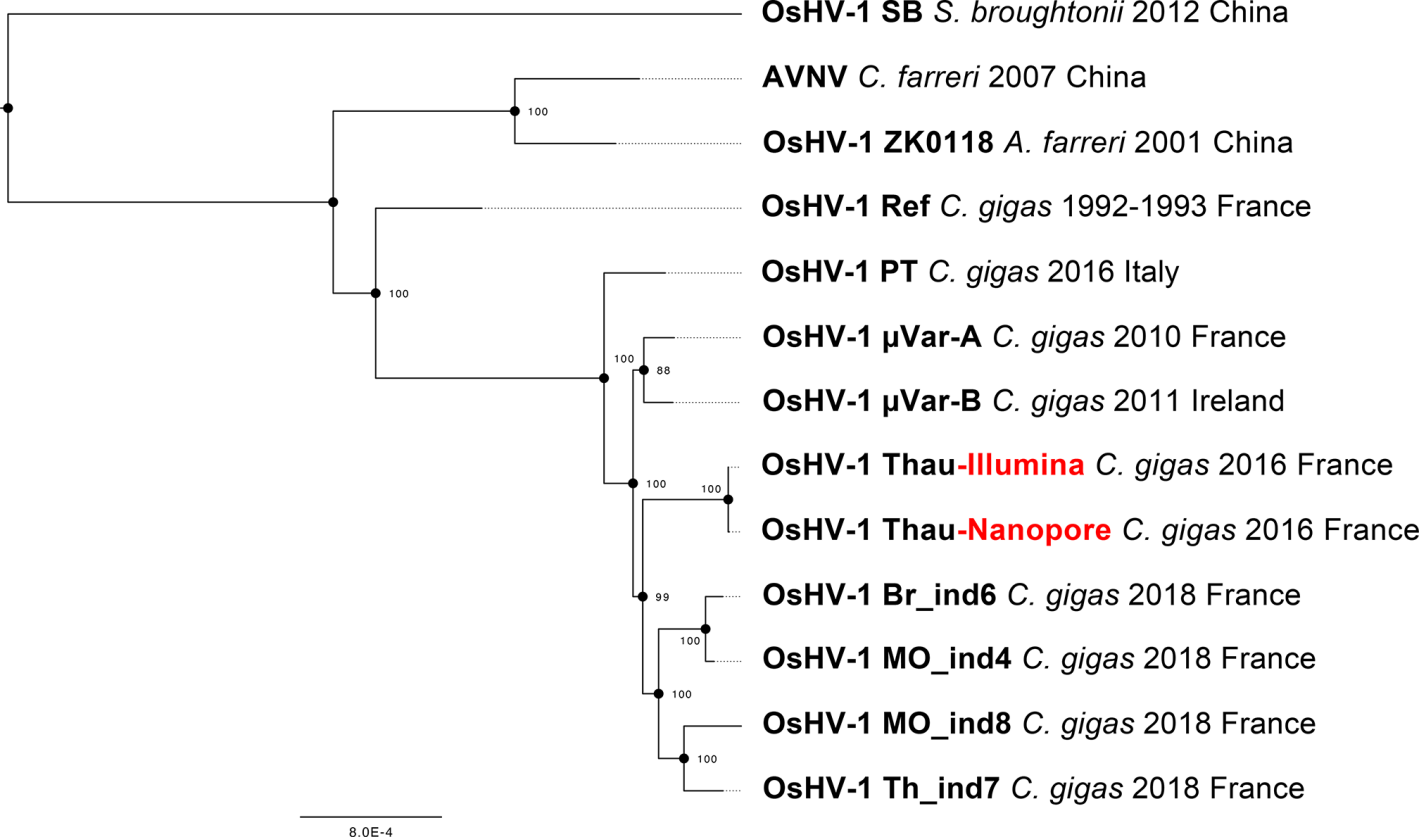
Phylogenetic tree of the 11 OsHV-1 NR version of the genomes available in public database, plus the Nanopore and Illumina NR genomes generated in the present study (red). The tree was mid-point rooted. Bootstrap values are shown at nodes (100 replicates).

In order to determine the relationships of the OsHV-1 genomes from this study with those known so far, we constructed a phylogenetic tree from the alignment of the two genomes we generated in the present study (viruses collected in 2016), seven OsHV-1 genomes available in public databases (viruses collected before 2016) and four representatives of the 21 genomes acquired by Delmotte-Pelletier *et al.* [34] (viruses collected in 2018). This analysis confirms that the OsHV-1 virus sampled in Thau lagoon (France) in 2016 belongs to µVar clade and that genomes reconstructed from the same sample with either the Nanopore or the Illumina sequencing data are really close to each other (Fig. 8).

### Non-OsHV-1 contigs' classification

Interestingly, our TFF-based purification process led to the characterization of other viral populations present in oyster tissues. To obtain a global overview of this viral metagenome, we made an extensive taxonomic classification of the non-OsHV-1 contigs produced by both ONT and Illumina sequencing. While the Nanopore sequencing produced 22 non-OsHV-1 contigs (from 14 374 to 163 592 bp) (Fig. 4b, Table S2A), Illumina sequencing produced 222 non-OsHV-1 contigs with size greater than 10 kb (from 10 017 bp to 172 478 bp) (Fig. 4a, Table S2B). Among the 22 Nanopore contigs 14 were also detected in the Illumina non-OsHV-1 contigs (Table S2A). Nanopore identified 20 contigs coding for putative bacteriophages, 1 contig coding for a CRESS DNA virus and 1 contig coding for the *C. gigas* mitochondrial DNA. Illumina identified 202 contigs' coding for putative bacteriophages, 19 contigs with no hit and 1 contig coding for the *C. gigas* mitochondrial DNA (Table S2B).

## Discussion

Viral whole-genome sequencing has recently become a standard in molecular epidemiology and evolutionary genomics, however this approach can be challenging when implemented on DNA viruses with large and complex genomes such as Herpesviruses [95, 96]. Indeed, it is often difficult to purify these types of viruses in sufficient quantity to allow whole-genome sequencing and reconstruction. Moreover, whole-genome assembly is a real burden considering the viral complexity due to the presence of long inverted repeated regions and structural variants. This is even more challenging to achieve for uncultured viruses derived from clinical samples or from livestock.

We implemented TFF to purify and to concentrate OsHV-1. To this end, we tested TFF devices with different pore sizes and quantified the virus load and infectious capacity of each fraction. Our results showed that TFF device with a MWCO of 500 kDa was able to retain and to concentrate most the OsHV-1 infectious particles contained in the inoculum. Interestingly, viral load quantification by real-time PCR and epifluorescence microscopy were of the same order of magnitude suggesting that TFF not only concentrates the virus but also eliminates most of the free DNA in the starting inoculum as previously suggested by Loewe *et al.* [60]. This is supported by the tremendous increase of the signal (OsHV-1 reads) to noise (oyster reads) ratio obtained in the sequencing data. Indeed, more than 60 % of the sequenced reads belonged to OsHV-1 whereas, in previous work, the ratio obtained from infected tissues ranged from 0,01% to 13,21% [31, 34]. While other virus enrichment methods like PCR or capture-based approaches can reach up to 100 % of viral data, the TFF approach does not suffer from amplification or hybridization bias caused by sequence variation over time due to genomic divergence [97]. We believe this method will be very helpful to improve the quantification of infectious viral particles in samples from organisms for which there is no cell culture available. Indeed, for tissue samples, the quantification of viruses is mainly based on qPCR approaches, which quantify the genome copy number but not the number of infectious viral particles. Since infected tissues could contain viral genomes undergoing replication or non-canonical genomes [48], the quantification of genome packaged in viral particle reflect a more robust estimate of the true quantification of viable virion in the sample. In addition, we have shown that TFF is an alternative particle enrichment method to centrifugation-based purification. TFF has many advantages including low cost, transportability, rapid purification, and enrichment in infectious particles (i.e. 500 kDa for OsHV-1). We therefore demonstrate, that TFF is a powerful tool to purify unculturable viruses from clinical and environmental isolates.

Then, we performed a high-quality DNA extraction that allowed us to compare the capabilities of long- and short-read sequencing technologies (Illumina and Oxford Nanopore Technologies) to *de novo* assemble OsHV-1 whole genome.

To date and to our knowledge, there is no maintained bioinformatic pipeline for reference-free and *de novo* assembly of long DNA viruses with large inverted-repeat like OsHV-1 for either short- or long-read sequence. Usually, long- and short-read sequencing technologies are combined to *de novo* assemble Herpesvirus whole genomes [48, 98]. Conversely to what is usually done, we have successfully tested two bioinformatic pipelines to independently analyse the long- and short-read datasets, and to reference-free and *de novo* assemble two OsHV-1 whole genomes. The *de novo* assembly of the short-read pipeline produced five OsHV-1 contigs that have been manually scaffolded to produce a complete OsHV-1 genome. The *de novo* assembly of the long-read pipeline produced one large contig that has been manually curated to remove one extra duplicated region. Because of uncertainty with the short-read assembly to position duplicated regions relative to unique region, we have depicted the Illumina assembly as a Non-Redundant (NR) genome, with only one copy of each R_L_ and R_S_, as frequently done in the literature [34, 77, 78, 99]. In contrast, the long-read assembly resulted in a genome with both inverted repeats with the help of a minimal manual edit.

Pairwise comparison of the two assembled OsHV-1 genomes showed that the Nanopore genome was 99,98 % similar to its Illumina counterpart. Our results demonstrate that Nanopore sequencing is now a mature sequencing technology for the small-scale genomic characterization of difficult long DNA virus with large inverted repeat. This breakthrough sequencing technology coupled with TFF virus purification is now a promising solution to drastically increase the number of sequenced OsHV-1 genomes allowing in-depth molecular evolution and epidemiology characterization.

In addition to being accurate at the base level, ONT sequencing also allowed to resolve large-scale genomic variation. For the first time here, the Nanopore sequencing allowed the identification of one extra copy of a 115 bp multi-copy repetition inside the IR_S_ region that was not previously resolved by short-read approach. This long-read sequencing approach also confirmed the presence of the four major OsHV-1 isomers in viral particles for the first time since the acquisition of the first OsHV-1 genome [7]. Isomers are characterized by permutation of the unique regions U_L_ and U_S_ with respect to the IR_L_-X-IR_S_ fragment. Some of the long-read obtained with Nanopore sequencing were large enough to cover one part of the two unique regions allowing us to quantify and confirm that the different OsHV-1 isomers were present in a stoichiometric proportion among viral particles. Interestingly, this observation is different from the one made in herpes simplex virus 1 (HSV-1), where four isomers have also been characterized, but with variable tropisms specific to cell lines and tissues [100]. Since the presence of isomeric genome in Herpesvirus is tightly linked to its replication and recombination process [98, 101, 102], having access to isomer frequency along with full genome characterization will allow in the near future a better understanding of the complex life cycle as well as of the evolutionary processes of this type of virus.

Finally, and even if this was not the primarily goal of this study, TFF allowed to co-purify multiple DNA viruses along with OsHV-1. For this specific application of viral metagenomics, Illumina allowed, due to the huge sequencing depth, to taxonomically identify ten times more virus than Nanopore sequencing for contigs with size larger than 10 kb. The great majority of identified DNA virus belong to bacteriophage. It is interesting to note that some of them are phages infecting *Vibrio *, bacteria, which have been described as colonizing moribund oysters during POMS outbreak [25, 103], others are Pseudoalteromonas and Aeromonas phages, pathogens of bacteria considered as beneficial for oysters [104, 105]. Of course, our data does not disentangle the potential role of these phages in POMS but raised questions about the necessity of investigating the virome of healthy and diseased oysters as it has been done for the bacteriome [25].

## Conclusion

This first evaluation of Nanopore sequencing for OsHV-1 genome characterization is extremely promising. Coupled with the appropriate bioinformatics pipeline this long-read sequencing approach enables identification of structural variation not observed with the short-read approach and allows characterization and quantification of genomic isomers. As shown in the present study, Nanopore sequencing still struggles to accurately sequence homopolymeric region. However, Nanopore is currently updating software for base calling as well as flow cell and chemistry to improve the resolution of the homopolymeric region [93, 106]. Illumina as well as Nanopore present difficulties to accurately resolve very long homopolymer (>20 bp) in GC-rich regions. It is thus of prime importance to focus efforts on methodological development for a better resolution of those regions that likely play a role for the diversity, replication and the stability of the viral genome [35, 107, 108].

The next challenge lies in combining TFF purification of virus populations harboured by a single individual and long-read sequencing to resolve viral haplotype phasing and better understand intra individual viral dynamics. Indeed, most of the whole-genome studies conducted on Herpesviruses to date only generate a consensus view of the viral population rather than a reconstruction of the viral haplotypes [96]. However, recent studies have shown that viral haplotype phasing could prove to be crucial in understanding the genetic determinants and viral evolution underpinning virulence, pathogenesis and drug resistance [109–111].

## Supplementary Data

Supplementary material 1
