# Supplementary material for: Evaluation of tangential flow filtration coupled to long-read sequencing for ostreid herpesvirus type 1 genome assembly
Source: Microb Genom. 2022 Nov 10;8(11):mgen000895. doi: 10.1099/mgen.0.000895 (PMC9836095; doi:10.1099/mgen.0.000895)
Supplement: Supplementary material 1 [file mgen-8-895-s001.pdf]

Figure Suppl 1

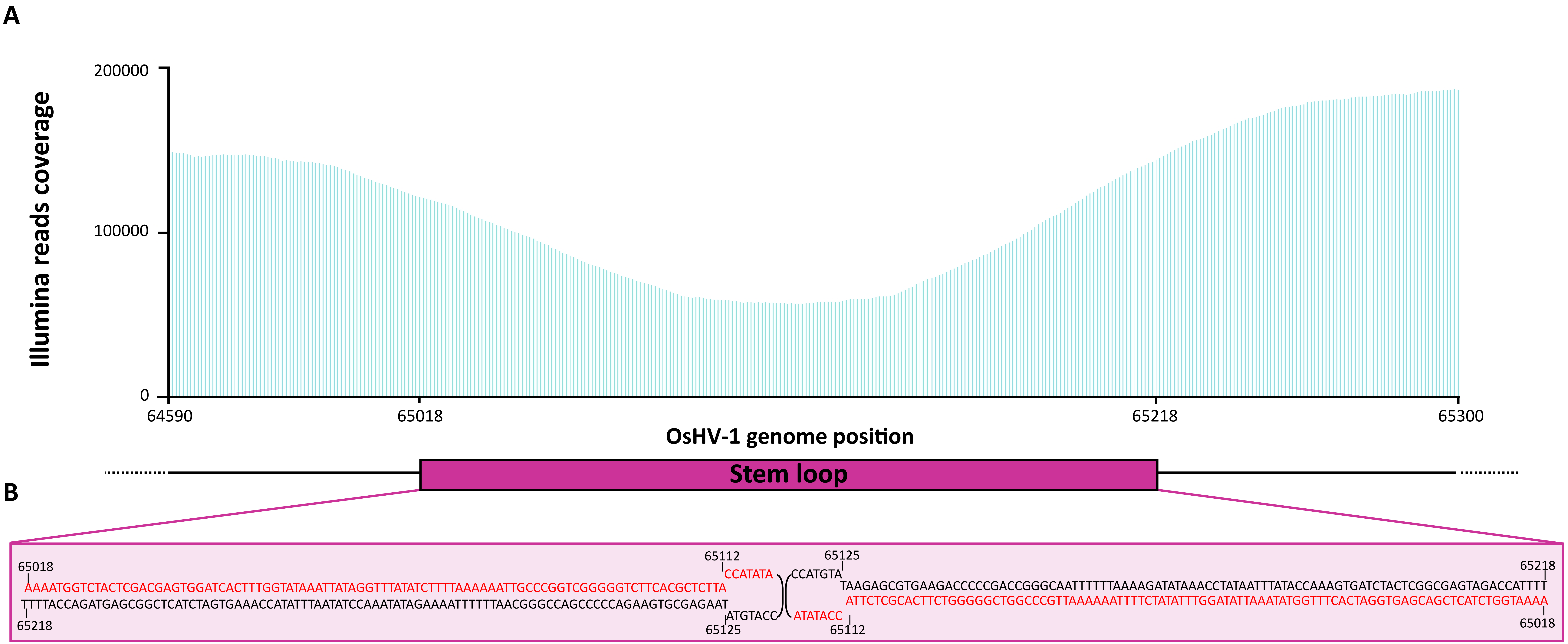

Figure Suppl 1: Read coverage analysis in the stem-loop region of the Illumina OsHV-1 NR genome.
(A) Illumina short-read coverage between position 64590 and 65300.
(B) Schematic representation of the palindromic region inside the stem-loop. Sequence color-coded in red correspond to the complementary strand forming the stem-loop.

Figure Suppl 2

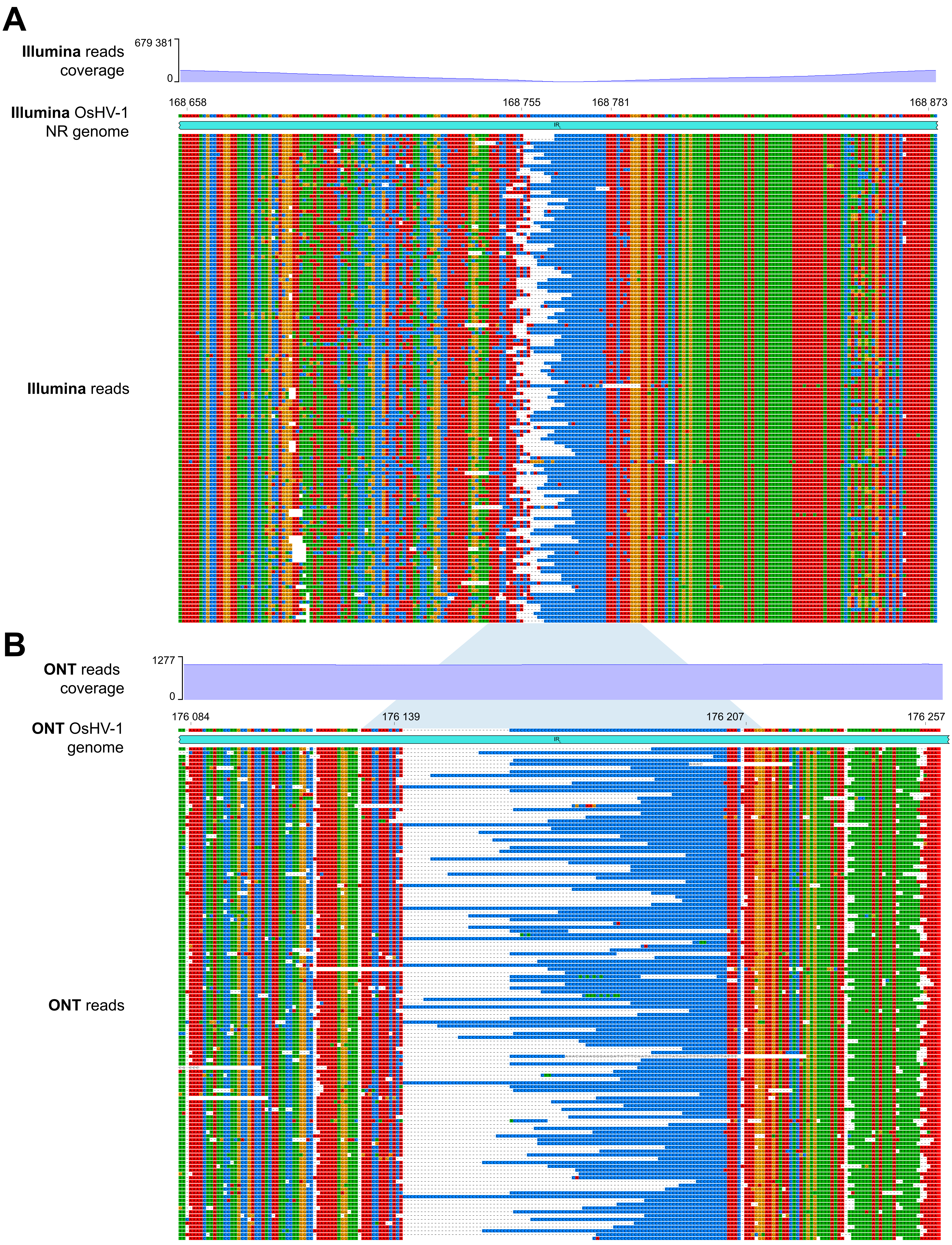

Figure Suppl 2: Large homopolymer read analysis.
(A) Snapshot of Illumina short-read aligned in the large homopolymer in the IRL region of the Illumina OsHV-1 NR genome.
(B) Snapshot of ONT long-read aligned in the large homopolymer in the IRL region of the ONT OsHV-1 genome.

Figure Suppl 3

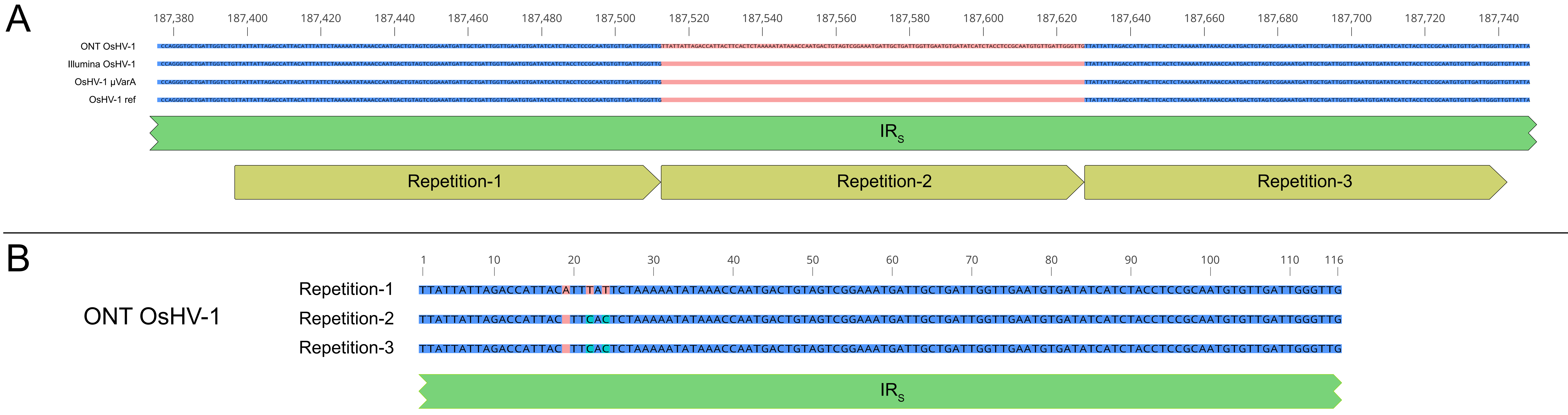

Figure Suppl 3: Schematic representation of the 115 bp structural variation present in the IRS region.

(A) Multiple alignment of the structural variation region from the 2005 OsHV-1 genome [7] the 2017 OsHV-1 µVar A genome [31], the Illumina OsHV-1 NR genome and the ONT OsHV-1 genome. In red is highlighted the missing repetition.

(B) Multiple alignment of the three copies of the repetition.

Table Suppl 1: Description of the methodologies used to date to sequence OsHV-1 genomes.

Reference	Strain	Genbank Accession	Publication Date	Origin	Host	Sampling Date	Tissus Type	Virus Isolation	DNA Extraction	% virus DNA (reads)	Amplification	Sequencing Librairy	Sequencing Technology	Assembly Type
Davison et al.	OsHV-1 Ref	AY509253	2005	France	*C. gigas*	1992-1993	Virions purified from infected *C. gigas* larvae	Ultra centrifugation + sucrose gradient	Phenol-chloroform	99%	/	M13mp19 library	Sanger	GCG / Ptrans / BLAST / FastA / PRED-TMR2
Ren et al.	AVNV	GQ153938	2013	China	*C. farreri*	2007	Virions purified from infected *C. farreri*	Ultra centrifugation + sucrose gradient	Takara MiniBEST DNA Extraction Kit Ver. 3.0	no data	Tiling PCR based on 2005 genome	pGEM-T	Sanger	DNASTAR
Xia et al.	OsHV-1 SB	KP412538	2015	China	*S. broughtonii*	2012	Virions purified from infected *S. broughtonii*	Ultra centrifugation + sucrose gradient	TIANampTM Marine Animals DNA Kit	no data	Tiling PCR based on 2005 genome	pEASYTM-T5 Zero Cloning Vector	Sanger	DNASTAR 7.1
Burioli et al.	OsHV-1 µVar A and B	KY242785 and KY271630	2017	France and Ireland	*C. gigas*	2010	Virions purified from infected *C. gigas* spat / minced spat tissues	Ultra centrifugation + sucrose gradient / no isolation	QIAamp DNA Mini kit	From ultracentrifugation + sucrose gradient = 1% to 66% (qPCR) / Tissus samples = no data	Pre-capture PCR for library enrichment	SPRIworks HT fragment library kit	Illumina® NextSeq® 500 system with a 2 × 150 bp Mid Output kit.	Reference-based (2005 genome) Bowtie2 / Samtools / MindTheGap
Abbadi et al.	OsHV-1 PT	MG561751	2018	Italy	*C. gigas*	2016	Gills and mantle from infected *C. gigas*	no isolation	QIAamp DNA Mini Kit	2,60%	Sequencing library amplification + Gap closing by PCR	Nextera XT DNA sample preparation kit	Illumina MiSeq + Sanger for finishing	Herpesvirales reads / denovo assembly IDBA-UD v1.1.1 / MUMmer v3.1 (2005 genome)
Bai et al.	OsHV-1 ZK0118	MF509813	2019	China	*C. farreri*	2001	Gills and mantle from infected *C. farreri*	no isolation	Qiagen DNeasy Blood and Tissue kit	no data	Tiling LR-PCR based on 2005 genome	9–13kb SMRTBell library / Illumina TruSeq	PacBio RS II / Illumina HiSeq 4000 / Sanger	PacBio: denovo Celera assembler + Quiver / Illumina: denovo SOAPdenovo + gapclose
Morga et al.	VIV46-2-m	PRJNA721248	2021	France, Charente-maritime	*C. gigas*	2017	Gills or mantle from infected spat-juveniles-adults C. gigas or pool of larvae	no isolation	QIAamp DNA Mini Kit	3,04%	/	/	HiSeq 4000, Paired-end 150bp	Reference mapping (2005 genome) BWA-MEM + consensus with bcftools and bedtools
	VIV56-10-m			France, Charente-maritime	*C. gigas*	2017				2,76%			HiSeq 4000, Paired-end 150bp	
	VIV48-4-m			France, Charente-maritime	*C. gigas*	2017				2,82%			HiSeq 4000, Paired-end 150bp	
	VIV49-5-m88			France, Charente-maritime	*C. gigas*	2017				1,92%			HiSeq 4000, Paired-end 150bp	
	VIV58-12-m			France, Charente-maritime	*C. gigas*	2017				1,19%			HiSeq 4000, Paired-end 150bp	
	VIV47-3-m			France, Charente-maritime	*C. gigas*	2017				3,19%			HiSeq 4000, Paired-end 150bp	
	VIV57-11-m99			France, Charente-maritime	*C. gigas*	2017				0,36%			HiSeq 4000, Paired-end 150bp	
	Pool-Harbour_UK_summer_2015			United-Kingdom	*C. gigas*	2015				3,53%			MiSeq, Paired-end 150bp	
	LI			France, Charente-maritime	*C. gigas*	2010				2,00%			HiSeq 2500, Paired-end 100bp	
	PR			France, Port en Bessin	*C. gigas*	2008				10,13%			HiSeq 2500, Single-end 100bp	
	MV			France, Charente-maritime	*C. gigas*	2010				11,62%			HiSeq 2500, Single-end 100bp	
	NZ			New-Zealand	*C. gigas*	2010				3,61%			HiSeq 2500, Single-end 100bp	
	NZ16			New-Zealand	*C. gigas*	2011				0,61%			HiSeq 2500, Paired-end 100bp	
	NZ17			New-Zealand	*C. gigas*	2011				0,88%			HiSeq 2500, Paired-end 100bp	
	NZ18			New-Zealand	*C. gigas*	2011				0,92%			HiSeq 2500, Paired-end 100bp	
	IRL15			Ireland	*C. gigas*	2011				0,01%			HiSeq 2500, Paired-end 100bp	
	JP2			Japan	*C. gigas*	na				0,01%			HiSeq 2500, Paired-end 100bp	
	JP6			Japan	*C. gigas*	na				0,01%			HiSeq 2500, Paired-end 100bp	
	NL4			Netherlands	*C. gigas*	na				0,02%			HiSeq 2500, Paired-end 100bp	
	SP16			Spain	*C. gigas*	na				0,01%			HiSeq 2500, Paired-end 100bp	
	SW3			Sweden	*O. edulis*	2012				0,01%			HiSeq 2500, Paired-end 100bp	
	SW6			Sweden	*O. edulis*	2012				2,12%			HiSeq 2500	
Delmotte et al.	OsHV-1 Br_ind2	OM811577	2022	France, Bay of Brest (Br) (Logonna-Daoulas, lat.: 48.335 long.: -4.318)	*C. gigas*	2018	Whole tissue from a single oyster spat	no isolation	MagAttract HMW DNA kit	1,35%	/	Shotgun PCR-free library preparation kit	Illumina NovaSeq 6000	Viral read extraction with Kraken and *C. gigas* reference mapping / Denovo assembly with SPAdes / Scaffolding with SSPACE using OsHV-1 µVar A as reference
	OsHV-1 Br_ind4	OM811578								1,73%				
	OsHV-1 Br_ind6	OM811579								1,09%				
	OsHV-1 Br_ind9	OM811580								1,10%				
	OsHV-1 Br_ind10	OM811581								3,10%				
	OsHV-1 MO_ind1	OM811582		France, Marennes-Oléron Bay (MO) (La Floride, lat.: 45.803 and long.: -1.153)						5,29%				
	OsHV-1 MO_ind3	OM811583								0,66%				
	OsHV-1 MO_ind4	OM811584								5,26%				
	OsHV-1 MO_ind7	OM811585								2,77%				
	OsHV-1 MO_ind8	OM811586								2,73%				
	OsHV-1 MO_ind9	OM811587								5,91%				
	OsHV-1 MO_ind10	OM811588								2,02%				
	OsHV-1 Th_ind1	OM811589		France, Thau Lagoon (Th) (Mèze, lat. 43.379 long. 3.571)						3,10%				
	OsHV-1 Th_ind3	OM811590								2,78%				
	OsHV-1 Th_ind4	OM811591								2,85%				
	OsHV-1 Th_ind5	OM811592								2,21%				
	OsHV-1 Th_ind6	OM811593								4,30%				
	OsHV-1 Th_ind7	OM811594								9,88%				
	OsHV-1 Th_ind8	OM811595								6,05%				
	OsHV-1 Th_ind9	OM811596								4,05%				
	OsHV-1 Th_ind10	OM811597								13,21%				

Table Suppl 2A: Taxonomic classification of non-OsHV-1 contigs. (A) Non-OsHV-1 contig identified in the ONT sequencing. Contig name in bold correspond to contigs also identified in the Illumina data.

Flye		Canu		Read Coverage (Flye all reads)	Lenght	GC%	Tool	Description	Accession
Name (all reads) (n=24)	Name (top10%) (n=9)	Name (all reads) (n=4)	Name (top10%) (n=5)						
**Contig_01**	Contig_05			6,4	41 032	52,9	PHASTER	Ralstonia phage RSK1 DNA	NC_022915.1
**Contig_02**				3,2	39 124	33,6	PHASTER	Flavobacterium phage FpV4	NC_041872.1
**Contig_03**				7,1	35 245	43,9	PHASTER	Pseudomonas phage tf	NC_017971.2
**Contig_04**				11,8	18 738	49,5	PHASTER	Cellulophaga phage phi39:1	NC_021804.1
**Contig_05**				4	45 019	37,5	PHASTER	Pseudoalteromonas phage BS5	NC_031917.1
**Contig_06**				5,1	24 004	31,5	BlastX	Phage	
Contig_07				4,7	27 938	36,5	BlastN	Vibrio phage pVp-1	NC_019529.1
**Contig_08**				6,4	29 804	31,7	PHASTER	Persicivirga phage P12024L	NC_018272.1
Contig_09				6,2	27 288	48,1	BlastN	Vibrio phage 1.151.O._10N.222.46.B1	MG592520.1
**Contig_10**				5,4	25 934	47,5	PHASTER	Lentibacter virus vB_LenP_ICBM2	NC_048671.1
**Contig_15**				7,3	32 985	34,9	PHASTER	Cellulophaga phage phi38:1	NC_021796.1
Contig_16				5,7	28 718	39,9	PHASTER	Flavobacterium phage vB_FspS_hemulen6-1	NC_048833.1
**Contig_17**	Contig_06	tig00000003		65,4	41 592	42	PHASTER	Vibrio phage pYD21-A	NC_020846.1
**Contig_18**	Contig_04			9,9	18 201	36,6	BlastN	C. gigas mitochondrial DNA	AF177226.1
Contig_19	Contig_12			5	64 902	67,5	PHASTER	Gordonia phage Bachita	NC_030936.1
**Contig_21**				4,1	35 292	44,8	PHASTER	Pseudoalteromonas phage Pq0	NC_029100.1
Contig_22		tig00000001		182,8	14 374	37	BlastN	Eukaryotic Circular Rep-Encoding Single-Stranded DNA (CRESS DNA) Viruses	MH617489.1
Contig_23				3,6	34 661	42,1	BlastN	Myoviridae sp. isolate ctZ7O1	BK025998.1
Contig_25	Contig_09			30,3	59 544	44,6	PHASTER	Vibrio phage douglas 12A4	NC_021068.1
**Contig_26**	Contig_10			22,6	163 592	33	PHASTER	Bacteroides phage crAss001	NC_049977.1
**Contig_27**	Contig_11			8,6	51 757	47,8	PHASTER	Vibrio phage VpKK5	NC_026610.2
Contig_28				4,9	29 738	43,3	BlastN	Uncultured Caudovirales phage genome assembly	LR798326.1

Table Suppl 2A: Taxonomic classification of non-OsHV-1 contigs. (B) Non-OsHV-1 contig identified in the Illumina sequencing.

Name	Contig FLYE (all reads)	Lenght	GC%	Tool	Description	Accession
NODE_1_length_172478_cov_93.767560		172 478	27.4%	PHASTER	Clostridium phage c-st	NC_007581
NODE_2_length_163260_cov_130.581300	**Contig_26**	163 260	33.0%	PHASTER	Bacteroides phage crAss001	NC_049977
NODE_3_length_130243_cov_16.614611		130 243	40.5%	PHASTER	Aeromonas phage 4_L372XY	NC_048772
NODE_4_length_125511_cov_20.062636		125 511	29.2%	BlastX	Phage	
NODE_5_length_124138_cov_147.751360		124 138	29.5%	PHASTER	Tenacibaculum phage PTm1	NC_049340
NODE_6_length_107092_cov_35.175883		107 092	29.6%	PHASTER	Cellulophaga phage phi4:1	NC_021788
NODE_7_length_100683_cov_47.882975		100 683	33.9%	BlastX	Vibrio phage	
NODE_8_length_100493_cov_10.918547		100 493	25.5%	PHASTER	Cellulophaga phage phi14:2	NC_021806
NODE_9_length_100113_cov_84.439085		100 113	31.5%	PHASTER	Cellulophaga phage phi14:2	NC_021806
NODE_11_length_98452_cov_33.513491		98 452	29.5%	PHASTER	Alteromonas phage vB_AmaP_AD45-P1	NC_021532
NODE_12_length_91848_cov_87.354014	**Contig_02**	91 848	32.5%	PHASTER	Flavobacterium phage Fpv1	NC_031914
NODE_13_length_91516_cov_52.058681		91 516	35.5%	BlastX	CrAssphage	
NODE_14_length_86979_cov_12.579564		86 979	35.9%	PHASTER	Alteromonas phage vB_AmaP_AD45-P1	NC_021532
NODE_15_length_83404_cov_54.393226		83 404	39.1%	PHASTER	Alteromonas phage vB_AmaP_AD45-P1	NC_021532
NODE_16_length_80651_cov_57.088491		80 651	44.4%	PHASTER	Iodobacteriophage phiPLPE	NC_011142
NODE_17_length_77861_cov_82.234879		77 861	31.9%	BlastX	Phage	
NODE_18_length_75926_cov_168.784358		75 926	45.3%	PHASTER	Caulobacter phage CcrBL9	NC_048047
NODE_19_length_71663_cov_12.848914		71 663	49.6%	PHASTER	Ruegeria phage vB_RpoP-V12	NC_049435
NODE_20_length_67566_cov_105.131993		67 566	32.2%	PHASTER	Bacteroides phage crAss001	NC_049977
NODE_21_length_65274_cov_85.326270		65 274	45.6%	PHASTER	Puniceispirillum phage HMO-2011	NC_021864
NODE_23_length_61469_cov_21.357541		61 469	49.1%	PHASTER	Salmonella phage vB_SenS_Sasha	NC_047786
NODE_24_length_60201_cov_26.626592		60 201	47.9%	PHASTER	Xanthomonas phage Xp15	NC_007024
NODE_25_length_53915_cov_55.201207		53 915	37.0%	PHASTER	Caulobacter phage CcrSC	NC_048048
NODE_26_length_50663_cov_14.441531		50 663	39.5%	PHASTER	Salinivibrio phage CW02	NC_019540
NODE_27_length_50338_cov_23.399360		50 338	32.6%	PHASTER	Riemerella phage RAP44	NC_019490
NODE_28_length_49661_cov_10.657662		49 661	37.9%	PHASTER	Cellulophaga phage phi14:2	NC_021806
NODE_29_length_48360_cov_61.567457		48 360	35.9%	PHASTER	Aeribacillus phage AP45	NC_048651
NODE_30_length_48330_cov_84.001264		48 330	43.2%	PHASTER	Pectobacterium phage ZF40	NC_019522
NODE_31_length_47526_cov_19.402920		47 526	47.1%	BlastX	Phage	
NODE_32_length_47107_cov_31.670173		47 107	37.8%	BlastX	Phage	
NODE_33_length_46830_cov_13.320791		46 830	53.0%	PHASTER	Sulfitobacter phage pCB2047-C	NC_020856
NODE_34_length_46181_cov_15.394116		46 181	58.2%	PHASTER	Enterococcus phage phiFL4A	NC_013644
NODE_35_length_46166_cov_11.843595		46 166	58.2%	PHASTER	Pelagibacter phage HTVC010P	NC_020481
NODE_36_length_46060_cov_26.568460	**Contig_15**	46 060	37.3%	PHASTER	Persicivirga phage P12024L	NC_018272
NODE_37_length_45467_cov_24.724148		45 467	43.7%	PHASTER	Proteus phage PM 116	NC_047858
NODE_38_length_44607_cov_87.013255		44 607	33.7%	PHASTER	Persicivirga phage P12024L	NC_018272
NODE_39_length_44415_cov_63.426375		44 415	38.3%	PHASTER	Alteromonas phage vB_AmaP_AD45-P1	NC_021532
NODE_40_length_43938_cov_12.537383		43 938	55.9%	PHASTER	Sulfitobacter phage pCB2047-A	NC_020858
NODE_41_length_43689_cov_12.105812		43 689	39.7%	PHASTER	Pseudoalteromonas Phage H103	NC_028819
NODE_42_length_42501_cov_39.647646		42 501	41.0%	PHASTER	Cronobacter phage phiES15	NC_018454
NODE_43_length_42044_cov_12.523661		42 044	28.1%	BlastX	Phage	
NODE_44_length_41271_cov_14.525136		41 271	59.5%	PHASTER	Sulfitobacter phage pCB2047-A	NC_020858
NODE_45_length_40813_cov_19.102753		40 813	27.7%	BlastX	Phage	
NODE_46_length_40564_cov_19.669950		40 564	57.9%	PHASTER	Escherichia phage ArgO145	NC_049918
NODE_47_length_40423_cov_14.034755		40 423	34.5%	PHASTER	Pseudomonas phage PA11	NC_007808
NODE_48_length_40353_cov_87.137153	**Contig_01**	40 353	52.7%	PHASTER	Sulfitobacter phage NYA-2014a	NC_027299
NODE_49_length_40321_cov_19.036880		40 321	51.8%	PHASTER	Ralstonia phage DU_RP_I	NC_047888
NODE_50_length_39832_cov_101.566383		39 832	31.8%	PHASTER	Polaribacter phage P12002S	NC_028763
NODE_51_length_39705_cov_24.240731		39 705	27.1%	BlastX	Phage	
NODE_52_length_39412_cov_12.145285		39 412	35.6%	PHASTER	Cellulophaga phage phi38:1	NC_021796
NODE_53_length_38773_cov_19.209980		38 773	55.8%	PHASTER	Pseudoalteromonas phage Pq0	NC_029100
NODE_54_length_37430_cov_12.030020		37 430	29.2%	BlastX	Phage	
NODE_55_length_37099_cov_77.038198		37 099	42.1%	PHASTER	Flavobacterium phage vB_FspS_snork6-1	NC_048840
NODE_56_length_36945_cov_43.883925		36 945	32.7%	PHASTER	Clostridium phage phiCD38-2	NC_015568
NODE_57_length_36621_cov_81.858065		36 621	30.9%	BlastX	Phage	
NODE_58_length_36610_cov_70.651648	**Contig_05**	36 610	37.2%	PHASTER	Pseudoalteromonas phage BS5	NC_031917
NODE_59_length_36510_cov_24.531532	**Contig_10**	36 510	45.9%	PHASTER	Celeribacter phage P12053L	NC_018280
NODE_60_length_36454_cov_13.185857		36 454	38.3%	PHASTER	Cellulophaga phage phi46:1	NC_021800
NODE_61_length_36388_cov_31.744089		36 388	40.2%	PHASTER	Thalassomonas phage BA3	NC_009990
NODE_62_length_35762_cov_143.499734		35 762	38.0%	PHASTER	Pseudoalteromonas phage BS5	NC_031917
NODE_63_length_35258_cov_116.610147	**Contig_05**	35 258	36.4%	PHASTER	Pseudoalteromonas phage BS5	NC_031917
NODE_64_length_35097_cov_167.852463	**Contig_21**	35 097	44.3%	PHASTER	Pseudomonas phage NV1	NC_042107
NODE_65_length_34770_cov_32.368241		34 770	32.5%	BlastX	Phage	
NODE_66_length_34723_cov_9.159455		34 723	33.0%	PHASTER	Polaribacter phage P12002S	NC_028763(
NODE_67_length_34634_cov_38.344776		34 634	27.4%	PHASTER	Cellulophaga phage phi17:2	NC_021798
NODE_68_length_34556_cov_25.344338		34 556	55.0%	PHASTER	Alteromonas phage vB_AcoS-R7M	NC_048878
NODE_69_length_34308_cov_43.308031		34 308	40.3%	PHASTER	Acinetobacter phage vB_AbaM_phiAbaA1	NC_031280
NODE_70_length_34211_cov_258.023363		34 211	44.7%	PHASTER	Escherichia phage Seurat	NC_027378
NODE_71_length_32957_cov_79.728740		32 957	32.8%	BlastX	Questionable phage	
NODE_72_length_31831_cov_25.768316		31 831	31.3%	BlastX	Phage	
NODE_73_length_31329_cov_20.645488		31 329	35.0%	PHASTER	Persicivirga phage P12024S	NC_018271
NODE_74_length_30831_cov_12.276774	**Contig_15**	30 831	37.4%	PHASTER	Pseudoalteromonas phage PH1	NC_031908
NODE_75_length_30800_cov_84.756741		30 800	30.1%	BlastX	Questionable phage	
NODE_76_length_30787_cov_11.777398		30 787	32.6%	PHASTER	Cellulophaga phage phi19:1	NC_021799
NODE_77_length_30471_cov_29.482969		30 471	36.8%	PHASTER	Vibrio phage PVA1	NC_023605
NODE_78_length_30421_cov_156.581901		30 421	29.7%	BlastX	Phage	
NODE_79_length_30315_cov_10.010542		30 315	31.1%	PHASTER	Dinoroseobacter phage DFL12phi1	NC_024367
NODE_80_length_29522_cov_26.494859		29 522	37.8%	PHASTER	Pelagibacter phage HTVC008M	NC_020484
NODE_81_length_29510_cov_10.884536		29 510	44.2%	PHASTER	Croceibacter phage P2559Y	NC_023614
NODE_82_length_28930_cov_49.046442	**Contig_15**	28 930	34.6%	PHASTER	Cellulophaga phage phi38:1	NC_021796
NODE_83_length_27924_cov_146.284725		27 924	29.3%	PHASTER	Tenacibaculum phage PTm1	NC_049340
NODE_84_length_27867_cov_97.891845		27 867	30.0%	PHASTER	Bacteroides phage crAss001	NC_049977
NODE_85_length_27453_cov_21.619826		27 453	55.5%	PHASTER	Roseobacter phage RDJL Phi 2	NC_041958
NODE_86_length_27411_cov_13.471633		27 411	37.5%	PHASTER	Bdellovibrio phage phi1422	NC_019525
NODE_87_length_27162_cov_11.561331		27 162	30.7%	PHASTER	Cellulophaga phage phi18:3	NC_021794
NODE_88_length_25739_cov_11.315683		25 739	37.8%	BlastX	Phage	
NODE_89_length_25673_cov_14.639004		25 673	41.6%	PHASTER	Pseudoalteromonas phage Pq0	NC_029100
NODE_90_length_25637_cov_10.846376		25 637	38.6%	PHASTER	Bacteroides phage crAss001	NC_049977
NODE_91_length_24325_cov_37.489617		24 325	38.6%	BlastX	Phage	
NODE_92_length_24017_cov_11.210208		24 017	44.2%	PHASTER	Lelliottia phage phD2B	NC_025450
NODE_93_length_23951_cov_11.148184		23 951	32.0%	BlastX	No phage hit	
NODE_94_length_23653_cov_11.141114		23 653	32.4%	PHASTER	Burkholderia phage Bcep176	NC_007497
NODE_95_length_23430_cov_13.129241		23 430	32.3%	BlastX	Phage	
NODE_96_length_22942_cov_132.097129		22 942	30.4%	PHASTER	Croceibacter phage P2559Y	NC_023614
NODE_97_length_22864_cov_42.207024		22 864	32.5%	BlastX	Phage	
NODE_98_length_22696_cov_46.953271		22 696	28.8%	BlastX	No phage hit	
NODE_99_length_22314_cov_10.365830		22 314	45.9%	PHASTER	Vibrio phage SHOU24	NC_023569
NODE_100_length_22222_cov_19.847792		22 222	33.8%	BlastX	Phage	
NODE_101_length_21801_cov_25.244413		21 801	35.4%	PHASTER	Dinoroseobacter phage vB_DshS-R5C	NC_041921
NODE_102_length_21781_cov_9.365000		21 781	30.7%	PHASTER	Clostridium phage phiCT9441A	NC_029022
NODE_103_length_21534_cov_10.542809		21 534	32.7%	BlastX	Phage	
NODE_104_length_21201_cov_71.793814		21 201	31.2%	BlastX	Questionable phage	
NODE_105_length_21069_cov_60.094461		21 069	31.3%	BlastX	Questionable phage	
NODE_106_length_20492_cov_12.357391		20 492	36.0%	BlastX	Phage	
NODE_107_length_19763_cov_15.903187	**Contig_01**	19 763	53.5%	PHASTER	Ralstonia phage RSK1	NC_022915
NODE_108_length_19636_cov_72.513865		19 636	30.0%	BlastX	Questionable phage	
NODE_109_length_19316_cov_78.317948		19 316	30.7%	PHASTER	Klebsiella phage ST147-VIM1phi7.1	NC_049451
NODE_110_length_19292_cov_123.286843		19 292	33.1%	BlastX	Phage	
NODE_111_length_19193_cov_84.839273	**Contig_27**	19 193	47.6%	PHASTER	Idiomarinaceae phage 1N2-2	NC_025439
NODE_112_length_19191_cov_12.705059		19 191	31.6%	PHASTER	Flavobacterium phage vB_FspS_tooticki6-1	NC_048844
NODE_113_length_19171_cov_13.206319	**Contig_15**	19 171	40.3%	PHASTER	Cellulophaga phage phi38:1	NC_021796
NODE_114_length_19062_cov_54.373547	**Contig_06**	19 062	30.6%	PHASTER	Faecalibacterium phage FP_Brigit	NC_047909
NODE_115_length_18774_cov_9.886532		18 774	50.1%	BlastX	Phage	
NODE_116_length_18724_cov_10.662703		18 724	39.4%	BlastX	Phage	
NODE_117_length_18614_cov_11.659464	**Contig_15**	18 614	33.5%	PHASTER	Cellulophaga phage phi38:1	NC_021796
NODE_118_length_18281_cov_1107.558927	**Contig_18**	18 281	36.6%	BlastN	C. gigas mitochondrial DNA	AF177226.1
NODE_119_length_17921_cov_16.542651		17 921	48.9%	BlastX	Phage	
NODE_120_length_17900_cov_46.778762		17 900	41.4%	PHASTER	Vibrio phage qdvp001	NC_029057
NODE_121_length_17850_cov_68.677775		17 850	34.1%	BlastX	No phage hit	
NODE_122_length_17689_cov_12.259896		17 689	38.8%	BlastX	No phage hit	
NODE_123_length_17415_cov_2993.154493	**Contig_17**	17 415	43.4%	PHASTER	Salicola phage CGphi29	NC_020844
NODE_124_length_17388_cov_19.204812		17 388	56.1%	PHASTER	Agrobacterium phage 7-7-1	NC_019519
NODE_125_length_17375_cov_10.886143		17 375	35.1%	PHASTER	Flavobacterium phage 11b	NC_006356
NODE_126_length_17189_cov_23.639314		17 189	39.4%	BlastX	Phage	
NODE_127_length_17169_cov_89.791516		17 169	33.3%	BlastX	No phage hit	
NODE_128_length_17151_cov_10.109850		17 151	36.2%	PHASTER	Ruegeria phage vB_RpoP-V13	NC_049436
NODE_129_length_16812_cov_98.559169		16 812	24.5%	BlastX	No phage hit	
NODE_130_length_16510_cov_121.542388		16 510	31.0%	BlastX	Questionable phage	
NODE_131_length_16487_cov_8.991176		16 487	38.7%	BlastX	Phage	
NODE_132_length_16481_cov_80.649093		16 481	32.7%	BlastX	Phage	
NODE_133_length_16416_cov_11.233115		16 416	30.4%	BlastX	Phage	
NODE_134_length_16303_cov_78.610906		16 303	32.7%	BlastX	Questionable phage	
NODE_136_length_16037_cov_62.140533	**Contig_03**	16 037	44.5%	PHASTER	Pelagibacter phage HTVC008M	NC_020484
NODE_137_length_15831_cov_13.061232		15 831	53.3%	PHASTER	Dinoroseobacter phage vB_DshS-R5C	NC_041921
NODE_138_length_15722_cov_60.445522		15 722	30.7%	BlastX	No phage hit	
NODE_139_length_15674_cov_136.733402		15 674	29.4%	BlastX	Phage	
NODE_140_length_15638_cov_7.874543		15 638	48.2%	BlastX	Phage	
NODE_141_length_15562_cov_10.017605		15 562	38.0%	PHASTER	Cellulophaga phage phi14:2	NC_021806
NODE_142_length_15558_cov_10.003161		15 558	32.5%	BlastX	Phage	
NODE_143_length_15525_cov_28.773432		15 525	41.2%	PHASTER	Vibrio phage CHOED	NC_023863
NODE_144_length_15386_cov_10.769552		15 386	47.2%	PHASTER	Rhizobium phage 16-3	NC_011103
NODE_145_length_15257_cov_9.517958		15 257	34.2%	BlastX	Phage	
NODE_146_length_15204_cov_146.422668		15 204	31.6%	BlastX	Phage	
NODE_147_length_15198_cov_35.388034		15 198	31.1%	PHASTER	Bacteroides phage crAss001	NC_049977
NODE_148_length_14711_cov_10.910139		14 711	32.5%	BlastX	Phage	
NODE_149_length_14655_cov_79.977534		14 655	31.6%	BlastX	No phage hit	
NODE_150_length_14635_cov_8.361180		14 635	31.6%	BlastX	Phage	
NODE_151_length_14505_cov_9.507820		14 505	46.4%	PHASTER	Salmonella phage UPF_BP1	NC_047875
NODE_152_length_14480_cov_29.114731		14 480	48.4%	PHASTER	Rhizobium phage 16-3	NC_011103
NODE_153_length_14411_cov_13.110546		14 411	34.7%	BlastX	Phage	
NODE_154_length_14367_cov_23.687814		14 367	56.9%	PHASTER	Roseobacter phage RDJL Phi 2	NC_041958
NODE_155_length_14322_cov_8.457489		14 322	57.4%	BlastX	Phage	
NODE_156_length_14131_cov_10.558610		14 131	37.7%	PHASTER	Clostridium phage phiCTP1	NC_014457
NODE_157_length_13958_cov_18.253974		13 958	55.5%	BlastX	Phage	
NODE_158_length_13942_cov_31.745803		13 942	32.9%	BlastX	Phage	
NODE_159_length_13801_cov_49.328168		13 801	30.7%	BlastX	No phage hit	
NODE_160_length_13779_cov_80.895220		13 779	34.3%	BlastX	No phage hit	
NODE_161_length_13779_cov_10.622268		13 779	48.9%	BlastX	Phage	
NODE_162_length_13760_cov_9.478511		13 760	32.1%	BlastX	Questionable phage	
NODE_163_length_13758_cov_157.247610		13 758	29.3%	BlastX	Phage	
NODE_164_length_13703_cov_11.308397		13 703	33.6%	BlastX	No phage hit	
NODE_165_length_13656_cov_10.734725		13 656	41.5%	PHASTER	Puniceispirillum phage HMO-2011	NC_021864
NODE_166_length_13649_cov_82.703031		13 649	31.9%	PHASTER	Pseudomonas phage PaMx74	NC_028809
NODE_167_length_13640_cov_10.671108		13 640	42.8%	PHASTER	Lentibacter virus vB_LenP_ICBM2	NC_048671
NODE_168_length_13607_cov_74.069436		13 607	32.6%	BlastX	Questionable phage	
NODE_169_length_13594_cov_12.991137		13 594	31.3%	BlastX	No phage hit	
NODE_170_length_13444_cov_75.132870		13 444	31.0%	BlastX	No phage hit	
NODE_171_length_13350_cov_2694.992854	**Contig_17**	13 350	41.4%	PHASTER	Idiomarinaceae phage Phi1M2-2	NC_025471
NODE_172_length_13233_cov_9.262559		13 233	30.9%	BlastX	No phage hit	
NODE_173_length_13174_cov_90.006403	**Contig_27**	13 174	46.4%	BlastX	Phage	
NODE_174_length_12972_cov_55.484942		12 972	28.4%	BlastX	No phage hit	
NODE_175_length_12937_cov_64.270300	**Contig_08**	12 937	32.3%	PHASTER	Persicivirga phage P12024L	NC_018272
NODE_176_length_12863_cov_9.662555		12 863	33.8%	BlastX	Phage	
NODE_177_length_12803_cov_8.331660		12 803	49.8%	PHASTER	Pseudoalteromonas Phage H103	NC_028819
NODE_178_length_12776_cov_9.605691		12 776	54.0%	PHASTER	Sulfitobacter phage NYA-2014a	NC_027299
NODE_179_length_12772_cov_8.876543		12 772	32.6%	PHASTER	Streptomyces phage phiELB20	NC_041849
NODE_180_length_12246_cov_9.410303		12 246	52.5%	PHASTER	Pantoea phage vB_PagS_Vid5	NC_042120
NODE_181_length_12219_cov_8.590184		12 219	38.7%	PHASTER	Pseudomonas phage pf16	NC_041881
NODE_182_length_12156_cov_10.336666		12 156	33.0%	PHASTER	Dinoroseobacter phage vB_DshS-R5C	NC_041921
NODE_183_length_12139_cov_10.847484		12 139	38.8%	PHASTER	Cellulophaga phage phi38:1	NC_021796
NODE_184_length_12061_cov_56.702066		12 061	32.0%	BlastX	Questionable phage	
NODE_185_length_11983_cov_7.494802		11 983	31.3%	BlastX	Phage	
NODE_186_length_11652_cov_10.117962		11 652	44.6%	PHASTER	Pseudoalteromonas phage Pq0	NC_029100
NODE_187_length_11518_cov_53.695891	**Contig_04**	11 518	48.3%	BlastX	Phage	
NODE_188_length_11488_cov_6.633342		11 488	30.6%	BlastX	Phage	
NODE_189_length_11466_cov_9.092542		11 466	37.0%	PHASTER	Mycobacteriophage Wheeler	NC_022070
NODE_190_length_11452_cov_46.784856		11 452	31.7%	BlastX	No phage hit	
NODE_191_length_11445_cov_10.025461		11 445	45.0%	PHASTER	Ralstonia phage RsoP1EGY	NC_047946
NODE_192_length_11430_cov_6.489055		11 430	30.1%	BlastX	Questionable phage	
NODE_193_length_11376_cov_9.457468		11 376	47.2%	PHASTER	Burkholderia phage phi644-2	NC_009235
NODE_194_length_11318_cov_9.591672		11 318	29.6%	BlastX	Phage	
NODE_195_length_11274_cov_11.619930		11 274	43.7%	BlastX	Phage	
NODE_196_length_11250_cov_12.972130		11 250	42.3%	PHASTER	Pseudomonas phage tf	NC_017971
NODE_197_length_11238_cov_34.232049		11 238	32.6%	PHASTER	Synechococcus phage S-SKS1	NC_020851
NODE_198_length_11214_cov_8.472981		11 214	32.6%	PHASTER	Alteromonas phage vB_AmaP_AD45-P1	NC_021532
NODE_199_length_11121_cov_54.963221	**Contig_06**	11 121	33.9%	BlastX	Phage	
NODE_200_length_11107_cov_10.116540		11 107	51.5%	BlastX	Phage	
NODE_201_length_11050_cov_12.667121		11 050	52.2%	BlastX	Phage	
NODE_202_length_11008_cov_9.699169		11 008	33.7%	PHASTER	Cyanophage S-TIM5	NC_019516
NODE_203_length_10988_cov_9.447544		10 988	31.4%	PHASTER	Dinoroseobacter phage DS-1410Ws-06	NC_049371
NODE_204_length_10946_cov_9.278395		10 946	51.4%	PHASTER	Dinoroseobacter phage vB_DshS-R5C	NC_041921
NODE_205_length_10902_cov_7.684705		10 902	40.7%	PHASTER	Synechococcus phage ACG-2014f isolate Syn7803C90	NC_026927
NODE_206_length_10849_cov_10.686029		10 849	37.8%	BlastX	Phage	
NODE_207_length_10815_cov_88.148978		10 815	32.0%	BlastX	No phage hit	
NODE_208_length_10692_cov_35.718530		10 692	47.3%	BlastX	Phage	
NODE_209_length_10657_cov_7.904169		10 657	31.5%	BlastX	Questionable phage	
NODE_210_length_10583_cov_7.575798		10 583	41.3%	PHASTER	Cellulophaga phage phi38:1	NC_021796
NODE_211_length_10556_cov_97.608894		10 556	32.8%	PHASTER	Bacillus phage G	NC_023719
NODE_212_length_10539_cov_8.692961		10 539	37.1%	PHASTER	Persicivirga phage P12024S	NC_018271
NODE_213_length_10424_cov_10.097502		10 424	31.5%	BlastX	Questionable phage	
NODE_214_length_10310_cov_9.925597		10 310	46.3%	BlastX	Phage	
NODE_215_length_10298_cov_17.006053		10 298	31.1%	PHASTER	Phage MedPE-SWcel-C56	NC_047768
NODE_216_length_10291_cov_82.179953		10 291	32.2%	BlastX	No phage hit	
NODE_217_length_10289_cov_36.401505		10 289	31.0%	BlastX	Phage	
NODE_218_length_10280_cov_39.834719		10 280	29.7%	BlastX	Phage	
NODE_219_length_10279_cov_18.776506		10 279	31.7%	BlastX	No phage hit	
NODE_220_length_10256_cov_34.185570		10 256	37.2%	BlastX	Phage	
NODE_221_length_10210_cov_8.268735		10 210	39.2%	PHASTER	Pelagibacter phage HTVC008M	NC_020484
NODE_222_length_10209_cov_16.740102		10 209	48.7%	BlastX	Phage	
NODE_223_length_10075_cov_9.561976		10 075	45.9%	PHASTER	Vibrio phage SHOU24	NC_023569
NODE_224_length_10035_cov_14.804409		10 035	29.5%	PHASTER	Persicivirga phage P12024S	NC_018271
NODE_225_length_10017_cov_6.580406		10 017	29.9%	BlastX	Phage	
